# Porous Bi_2_S_3_ Bulk With Excellent Thermoelectric Performance by Solid States Replacement and Low Melting‐Point Metal Volatilization

**DOI:** 10.1002/adma.202521215

**Published:** 2026-01-02

**Authors:** Zi‐Yuan Wang, Jun Guo, Yi‐Xin Zhang, Hao Liang, Xing Yang, Rafal E. Dunin‐Borkowski, Fengshan Zheng, Lei Jin, Jing Feng, Zhen‐Hua Ge

**Affiliations:** ^1^ Faculty of Material Science and Engineering and National & Local Joint Engineering Laboratory of Advanced Metal Solidification Forming and Equipment Technology Kunming University of Science and Technology Kunming China; ^2^ Southwest United Graduate School Kunming China; ^3^ Ernst Ruska‐Centre For Microscopy and Spectroscopy with Electrons Forschungszentrum Jülich GmbH Jülich Germany; ^4^ Center for Electron Microscopy South China University of Technology Guangzhou China

**Keywords:** Bi_2_S_3_, FeCoNi doping, porous structures, thermoelectric materials

## Abstract

Bismuth sulfide (Bi_2_S_3_) exhibits potentials in thermoelectric field, due to their environmental friendliness, high Seebeck coefficients, and low thermal conductivity. However, the peak *ZT* for binary Bi_2_S_3_ does not exceed 1.0, inhibiting its practical applications. Starting from the precipitation smelting of bismuth concentrate process, this study constructs multi‐type, multi‐scale in‐situ secondary phases and porous structures through FeCoNi (FCN) medium‐entropy alloy addition, significantly enhancing the *ZT* value of Bi_2_S_3_‐based thermoelectric materials. The introduced FCN reacts with pre‐synthesized Bi_2_S_3_ nanorod matrix during spark plasma sintering and forms precipitate complex with FCN‐S core and Bi shell microstructures. FCN doping improves the carrier concentration of Bi_2_S_3_ and the reduced Bi from Bi_2_S_3_ acts as carrier transport channels for mobility optimization. Due to the stacking effect of Bi_2_S_3_ nanorods and the volatile nature of metallic Bi, porous Bi_2_S_3_ structure is formed, characterized by randomly‐distributed and micro‐to‐nanoscale pores. The coexistence of various lattice defects effectively scatter phonons and suppress the lattice thermal conductivity, thus an excellent peak *ZT* of 1.1 is achieved at 773 K in a 0.25 wt.% FCN‐doped Bi_2_S_3_ sample. This study, drawing on the process of ore smelting, proposes a convenient method for preparing high‐performance chalcogenide thermoelectric materials with porous structures.

## Graphical abstract

By introducing FeCoNi medium‐entropy alloy, the bismuth sulfide (Bi_2_S_3_) material achieves a record‐high ZT of 1.1 at 773 K, owing to the solid‐states replacement reaction and the volatilization of low melting‐point metal. This strategy is also applicable to other sulfur‐based thermoelectric materials.

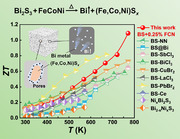

## 1 Introduction

Thermoelectric materials can directly convert waste heat into electricity using the Seebeck effect or convert electricity into heat using the Peltier effect without moving component parts or generating toxic gases, thus providing a new approach to overcome the increasingly serious environmental issues and energy crisis [1, 2, 3]. The performance of a thermoelectric material is evaluated using the dimensionless figure of merit *ZT* [4, 5, 6]. *ZT* = (*σS* ^2^/*κ*_tot_)*T*, where *σ*, *S*, *κ*_tot_, and *T* represent the electrical conductivity, the Seebeck coefficient, the total thermal conductivity, and the absolute temperature (in K), respectively.

Simultaneous optimization of the electrical and thermal transport properties is challenging due to the complex and strong coupling among the *σ*, *S*, and *κ*_tot_ parameters. Nevertheless, the power factor (*PF* = *σS* ^2^) can be effectively improved by increasing *σ* using carrier engineering [7, 8, 9] (modulation doping and elemental alloying for carrier density optimization) and by increasing *S* using band‐structure engineering [10, 11, 12] (enhancing band degeneracy and band convergence for effective mass optimization). In parallel, decreasing lattice thermal conductivity (*κ*_lat_) is also beneficial for enhancing the *ZT* value. Strategies have been developed to optimize thermal transport property, such as enhancing the phonon scattering through the introduction of nanostructure precipitates [13, 14, 15], utilizing porous structures [16, 17], or seeking new materials with intrinsically low thermal conductivities [18, 19, 20].

There are many state‐of‐the‐art thermoelectric materials with excellent thermoelectric performances, such as Bi_2_Te_3_ [21, 22, 23, 24], PbQ‐based (Q = S, Se, and Te) [25, 26, 27, 28], SnTe‐based [11, 29], and Half‐Heusler alloys [30, 31]. However, their large‐scale applications are still restricted due to the requirement of rare and expensive elements, such as Te, or toxic and carcinogenic elements, such as Pb, Cd, and Sb. As a consequence, it is critical to find and further optimize new candidates that contain earth‐abundant and environment‐friendly elements in order to boost further market applications. Recently, Pb‐free Bi_2_S_3_, consisting of abundant, cheap, and low‐toxic elements, has attracted increasing attention for medium‐temperature commercial applications. Bi_2_S_3_ has a room‐temperature (RT) orthorhombic structure, and the chemical bonds between Bi and S are more ionic than covalent. Because of the significantly large difference in electronegativities of Bi (= 1.9) and S (= 2.58), a strong optical phonon scattering is generated, resulting in a low *κ*_lat_ and poor carrier mobility [32]. The large *S* value and a relatively low *κ*_lat_ make Bi_2_S_3_ a promising medium‐temperature thermoelectric material. However, the low *σ* seriously inhibits its improved thermoelectric properties, as the intrinsic carrier concentration (*n*) of Bi_2_S_3_ is as low as 10^17^ cm^−3^. Previous studies have demonstrated that metal element doping (such as Ag [33, 34], Cu [35, 36], Ce [37]) can effectively modulate the carrier concentration and phonon scattering in Bi_2_S_3_‐based thermoelectric materials, thereby enhancing their performance. Meanwhile the hydrothermal method combined with spark plasma sintering (SPS) has also been employed to construct tailored microstructures for further optimization. For instance, Liu et al. [38] synthesized Bi_2_S_3_ nano‐networks via a solution‐based approach and achieved a *ZT* value of 0.5 at 723 K through grain boundary purification. Xu et al. [16] further engineered the stacking of Bi_2_S_3_ nano‐networks and introduced a porous structure via SPS, which significantly reduces *κ*_lat_ and achieves a peak *ZT* of 0.81 at 823 K. This highlights the synergistic enhancement of thermoelectric properties through microstructural design.

Introducing porous structures into bulk thermoelectric materials has been theoretically proven to effectively optimize the thermoelectric properties. Various strategies have been proposed to construct porous structures in bulk thermoelectric materials, including assembly of hollow nanostructures [39], chemical exfoliation [40], sublimating agent‐assisted pore formation [41], and explosive reaction methods [42]. However, the intense carrier/phonon scattering at the interface between the matrix material and pores may lead to simultaneous reductions in *σ* and *κ*_lat_. Since the decrease in *κ*_lat_ cannot fully compensate for the deterioration of *σ*, the *ZT* value may be eventually reduced. In this regard, designing the interface between the matrix and pores, as well as controlling the size and distribution of pores, is crucial for the performance improvements of thermoelectric materials.

**FIGURE 1:**
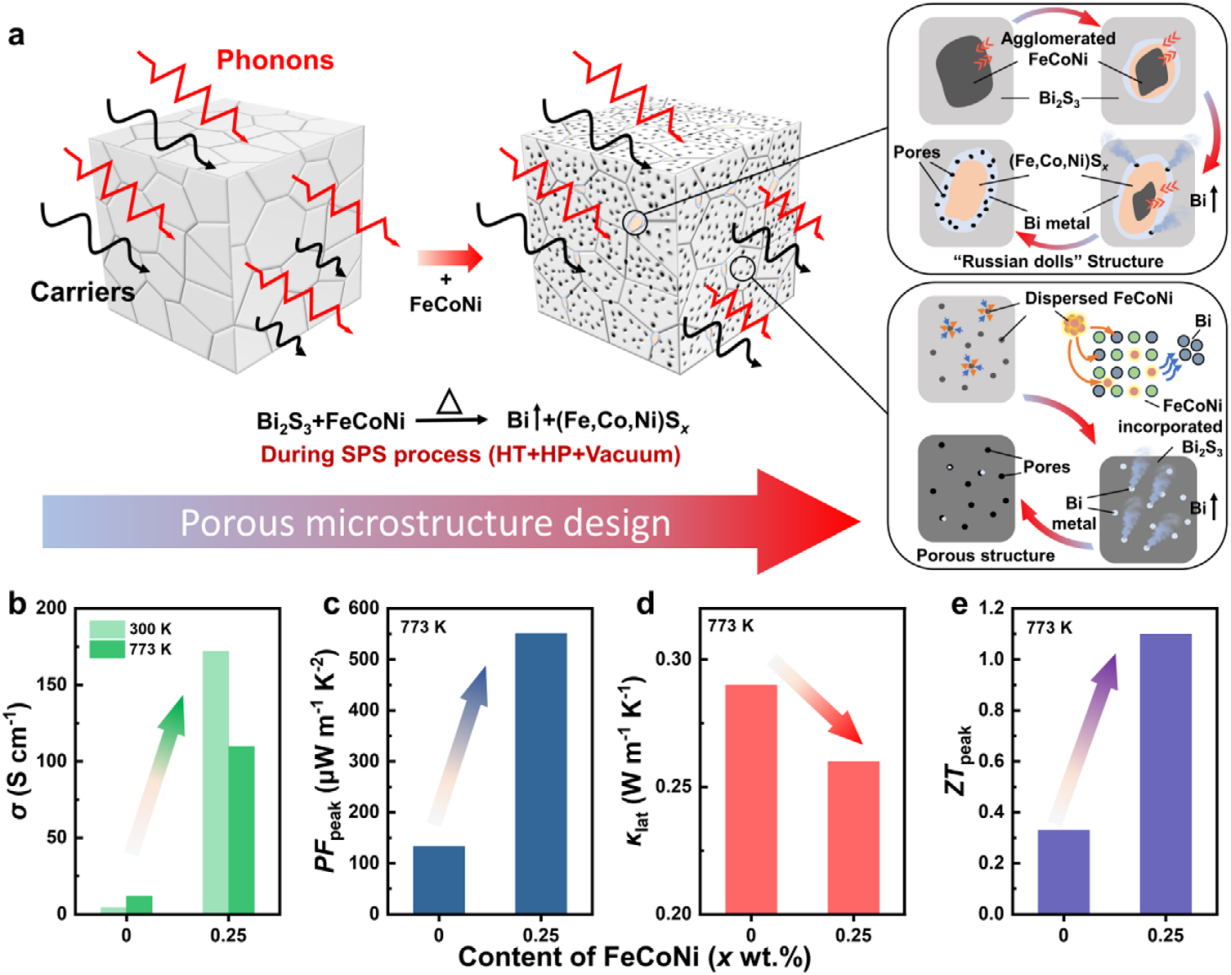
Schematic diagram of the Bi_2_S_3_ based bulk material with complex microstructure prepared by SPS of Bi_2_S_3_ nanorods and FeCoNi alloy nano‐powders. (a) The formation process of the porous structure. (b) Electrical conductivity, (c) peak power factor, (d) lattice thermal conductivity and (e) *ZT* of Bi_2_S_3_ and Bi_2_S_3_ + 0.25 wt.% FCN bulk samples.

In this work, hydrochloric acid‐pretreated Bi_2_S_3_ nanorods are mixed with iron‐based medium‐entropy alloy (FeCoNi, abbreviated as FCN) and Bi_2_S_3_‐based bulk polycrystalline materials are synthesized by SPS following the precipitation smelting process [43]. The synthetic details are shown in Figure S1. In the precipitation smelting process, FCN could react with Bi_2_S_3_ concentrate, generating metallic Bi and (Fe,Co,Ni)S*_x_* (FCN‐S) precipitates; meanwhile, FCN can also enter Bi_2_S_3_ lattice and achieve effective doping, as shown in Figure 1a. The reduced Bi forms around the FCN‐S compounds, resulting in a nested distribution of the multiple and multiscale precipitates. The *n* and the carrier mobility (*µ*) of Bi_2_S_3_ material are simultaneously improved due to the heterovalent FCN doping and the formation of conductive channels through the reduced Bi precipitates. Therefore, the highly enhanced *σ* and *PF* is realized (Figure 1b,c). Moreover, densely distributed and multi‐scale pores are formed (Figure 1a), which are induced by nanorod stacking and volatilization of the reduced Bi metals (under the high temperature (HT), high pressure (HP) and vacuum environmental conditions during SPS). The complex microstructures, consisting of metal sulfides and multi‐scale pores, could effectively scatter phonons with different frequencies, leading to an ultralow *κ*_lat_ (Figure 1d). Ultimately, an n‐type, binary, polycrystalline Bi_2_S_3_ + 0.25 wt.% FCN sample is obtained with an excellent *ZT* value of 1.1 at 773 K (Figure 1e), surpassing previous reports and demonstrating the potential of Bi_2_S_3_ materials for thermoelectric applications in the middle‐temperature region.

## 2 Results and Discussion

**FIGURE 2:**
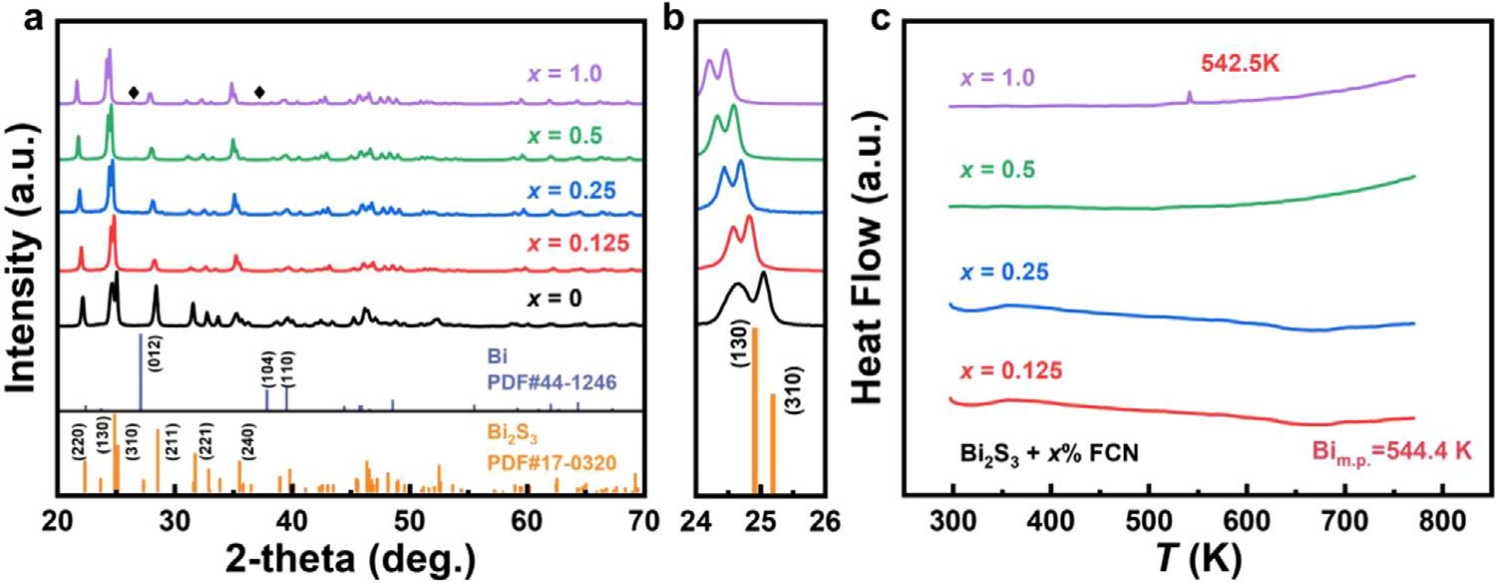
(a) XRD patterns and (b) expanded plots for the (130) and (310) peaks of Bi_2_S_3_ + *x* wt.% FCN (*x* = 0, 0.125, 0.25, 0.5, 1.0). (c) Differential scanning calorimeter results for all samples in the range of 300 – 800 K.

The X‐ray diffraction (XRD) patterns of the bulk Bi_2_S_3_ + *x* wt.% FCN (*x* = 0, 0.125, 0.25, 0.5, 1.0) samples are shown in Figure 2a. All featured peaks match well with the orthorhombic Bi_2_S_3_ phase. The enlarged XRD pattern for the diffraction angle between 24–26° is shown in Figure 2b. As *x* increases, the XRD peaks gradually shift toward lower angles, suggesting a systematic lattice expansion of the Bi_2_S_3_ matrix. The lattice expansion may be explained by the fact that the FCN ions enter the interstitial sites of Bi_2_S_3_. Volatilization of S during SPS may be another reason for the volume increase, as reported in literature [44]. Notably, the diffraction peaks corresponding to metallic Bi can be detected when the FCN content reaches 1.0 wt.%. Furthermore, the elemental Bi is further verified using differential scanning calorimetry for all samples in the 300 – 800 K temperature range, and the results are shown in Figure 2c. The Bi_2_S_3_ + 1.0 wt.% FCN sample shows an obvious absorption peak near 542.5 K, consistent with the melting point of metallic Bi at 544.4 K. Additionally, the thermogravimetry curves do not show a decreasing inflection point in the measured temperature range, indicating a good thermal stability of the prepared Bi_2_S_3_ samples, as shown in Figure S2.

**FIGURE 3:**
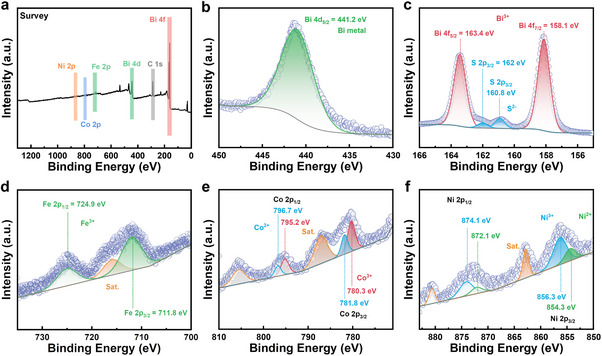
High resolution XPS spectra of the Bi_2_S_3_ + 0.25 wt.% FCN sample. (a) Survey spectrum and spectra of (b) Bi 4*d*, (c) Bi 4*f* and S 2*p*, (d) Fe 2*p*, (e) Co 2*p* and (f) Ni 2*p*.

As shown in Figure 3a, the chemical state of different elements on the surface of the as‐synthesized bulk samples is analyzed using X‐ray photoelectron spectroscopy (XPS). Here, Bi_2_S_3_ + 0.25 wt.% FCN is used as a typical example. As shown in Figure 3b, the peak at 441.2 eV corresponds to Bi 4*d*_5/2_ of the Bi metal [45], indicating the presence of Bi metal in this sample. Apparently, the amount is beyond the detection limit of XRD and DSC. The peaks at 158.1 and 163.4 eV can be attributed to the Bi 4*f*_7/2_ and Bi 4*f*_5/2_ orbits, respectively [46]. Additionally, as shown in Figure 3c, two minor peaks are detected at 160.8 and 162.0 eV in the same band area of the Bi 4*f* orbit, arising from S 2*p*_3/2_ and S 2*p*_1/2_ [47]. Consequently, the valence state of the matrix elements Bi and S are +3 and –2, respectively. In comparison with Bi and S, Fe, Co and Ni have weaker peak intensities, indicating that their contents are low on the measured surface. As shown in Figure 3d, the Fe^3+^ species are present, and the binding energies at 711.8 and 724.9 eV can be ascribed to Fe^3+^ 2*p*_3/2_ and Fe^3+^ 2*p*_1/2_ [48], respectively. Additionally, the peak with binding energy at 715.7 eV can be attributed to the satellite peak of Fe 2*p*. As shown in the Co 2*p* spectrum in Figure 3e, the main doublets at 781.8, 796.7, 780.3, and 795.2 eV correspond to Co^2+^ 2*p*_3/2_, Co^2+^ 2*p*_1/2_, Co^3+^ 2*p*_3/2_, and Co^3+^ 2*p*_1/2_, respectively [48]. As shown in the Ni 2*p* spectrum in Figure 3f, Ni^2+^ (854.3 for Ni^2+^ 2*p*_3/2_ and 872.1 eV for Ni^2+^ 2*p*_1/2_) and Ni^3+^ (856.3 for Ni^3+^ 2*p*_3/2_ and 874.1 eV for Ni^3+^ 2*p*_1/2_) coexist [49, 50]. Notably, the Fe, Co, and Ni metallic states cannot be detected in the Bi_2_S_3_ + *x* wt.% FCN sample, indicating that FCN reacts with the Bi_2_S_3_ matrix during SPS and forms compounds.

**FIGURE 4:**
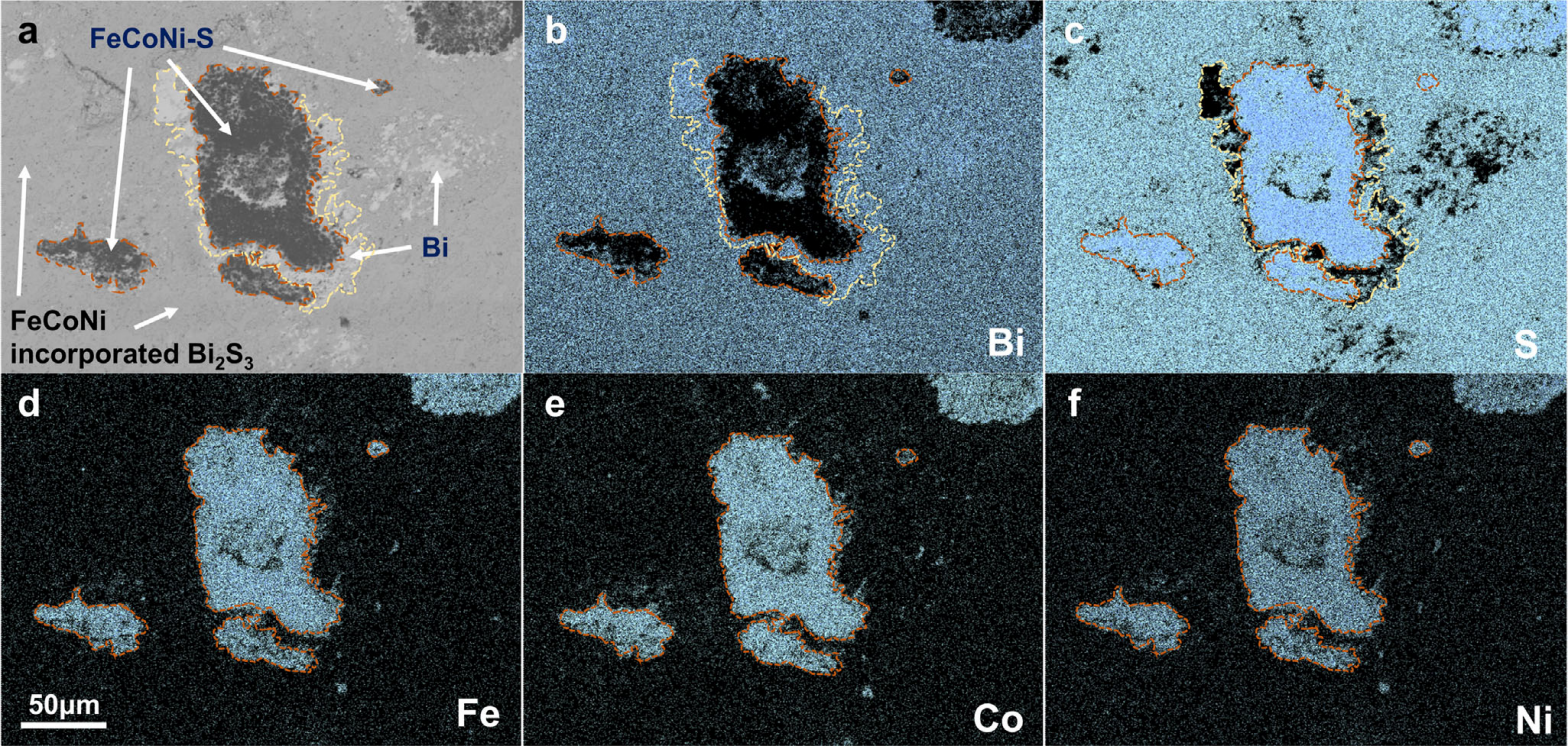
(a) BSE SEM image of the Bi_2_S_3_ + 0.25 wt.% FCN sample. The sample surface is polished for measurements. EPMA elemental mapping result of (b) Bi, (c) S, (d) Fe, (e) Co and (f) Ni element in the Bi_2_S_3_ + 0.25 wt.% FCN sample. The results are obtained from identical locations, therefore a global scale bar is displayed in (d).

The backscattered electron (BSE) scanning electron microscopy (SEM) image shown in Figure 4a reveals three different contrasts (i.e., grey, black and white) in the Bi_2_S_3_ + 0.25 wt.% FCN sample. Same contrasts are also observed in other FCN treated samples, as shown in Figure S3. The elemental distribution results obtained by electron probe micro analysis (EMPA) reveal that the gray area is the Bi_2_S_3_ matrix incorporated by a small amount of Fe, Co and Ni (denote FCN incorporated Bi_2_S_3_, see also Figure S4 and Table S1). The distribution of Fe, Co and Ni is found to be uniform, which is beneficial to the overall material conductivity. The black area is primarily enriched with Fe, Co, Ni and S elements, indicating the formation of FCN‐S compounds. This is consistent with the XPS results. The density of this FCN‐S phase increases as the FCN content is increased, as evidenced in Figure S3. The white region is metallic Bi, which is formed as a result of the reduction reaction between FCN and Bi_2_S_3_. Notably, the polymorphic secondary phases form a multilayer “Russian dolls” structure, usually with metal sulfide as the core and metallic Bi as the surrounding layer. Their size can be varied from several to hundreds of microns, which imposes difficulties for scanning transmission electron microscopy (STEM) investigations. Nevertheless, a small‐scale “Russian doll” structure is captured in Figure S5 and the energy dispersive X‐ray spectroscopy (EDS) results are consistent with EPMA. Such complex microstructures introduce additional phase boundaries, which is expected to reduce *κ*_lat_.

**FIGURE 5:**
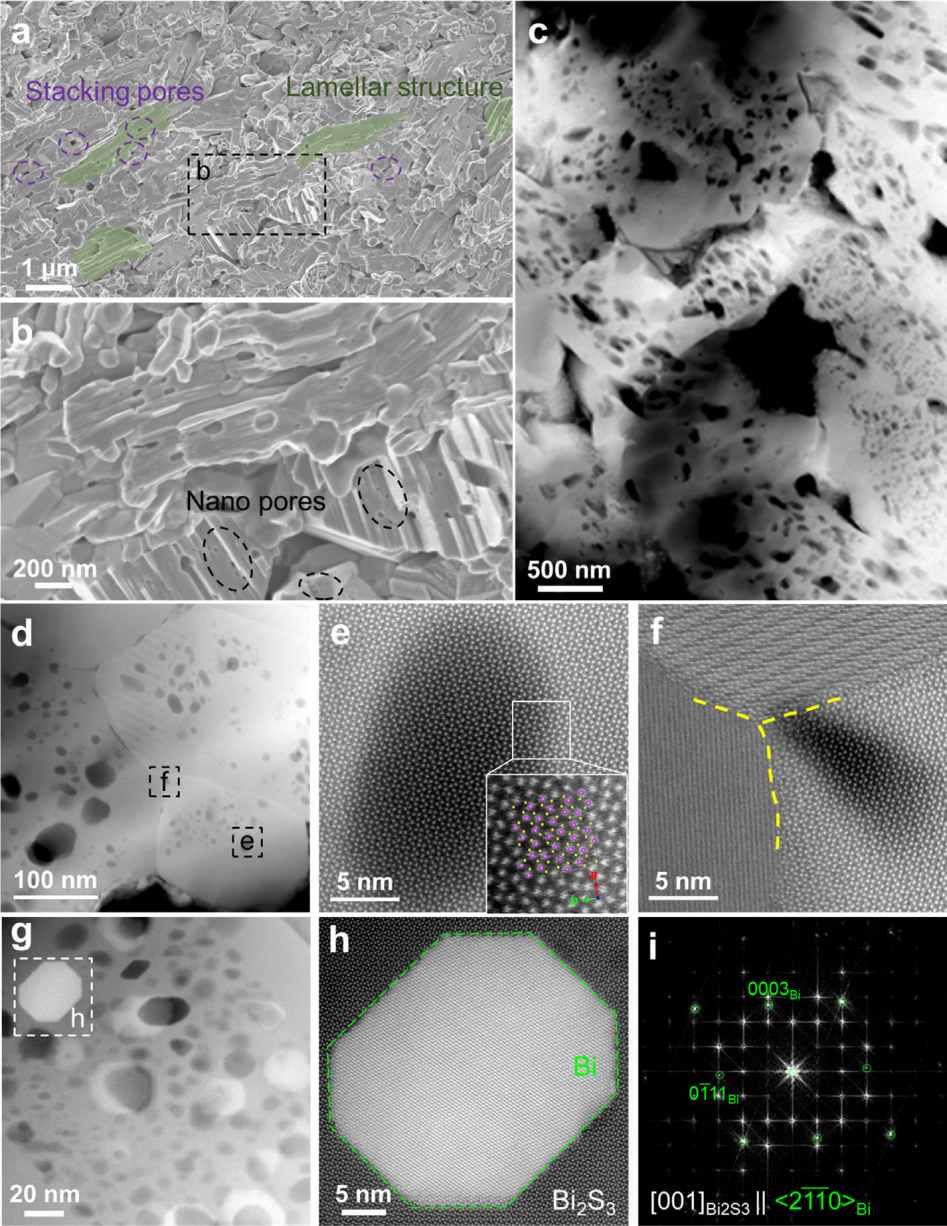
(a) SEM of Bi_2_S_3_ + 0.25 wt.% FCN sample showing the formation of lamellar structure accompanied by the stacking related pores. (b) Magnified SEM from the marked area in (a) showing the presence of nanopores in grains. (c) and (d) High‐angle annular dark‐field scanning transmission electron microscopy (HAADF‐STEM) images of Bi_2_S_3_ + 0.25 wt.% FCN sample at different magnifications. (e) and (f) Atomic‐resolution HAADF images of the areas marked in (d). (g) Low magnification HAADF image showing the coexistence of residual Bi and nanopores; (h) Magnified HAADF image of the area marked in (g) and (i) the corresponding fast Fourier transform showing the relationship between Bi and the grain matrix.

The fracture surface morphologies of the Bi_2_S_3_ + *x* wt.% FCN samples are studied using SEM, as shown in Figure 5a,b and Figure S6. It is evidenced that FCN treatment significantly affects the microstructures, as compared with that of the Bi_2_S_3_ pure sample shown in Figure S7. On the one hand, the nanorod morphology is reduced with increased FCN treatment. We note that FCN has a high electrical conductivity (7000 – 8000 S cm^−1^) [51], it increases the local current density during SPS. This creates HT spots and leads to local overheating, thus reshaping the grain morphology. On the other hand, the average Bi_2_S_3_ grain size decreases from 600 to 400 nm as FCN content increases from 0.125 to 1.0 wt.%, as shown in Figure S6. This can be interpreted by the fact that the increase in FCN content increases the HT spots. In addition, the presence of hard secondary phases (FCN‐S) may also hinder the grain growth during sintering. With the abovementioned reasons, a typical laminar structure is formed and accompanying the nanorod stacking, pores are also evident, as shown in Figure 5a. The size of these stacking related pores is measured for the pure and *x* = 0.25 samples, as shown in Figure S8. With FCN reaction, the size is reduced suggesting a better grain connectivity and structural densification. This is evidenced by the increase of relative densities as a function of *x*, measured by Archimedes method (Table S2).

The reaction of FCN not only introduces multi‐scale “Russian dolls” structure into the matrix, but also leads to the formation of abundant nanoporosity within the matrix grains, creating a nanoporous structure. Unlike the stacking induced pores (with larger pore size), these nanopores are less than 50 nm as evidenced by the magnified SEM image shown Figure 5b. In order to further understand the microstructures, we perform STEM together with EDS.

Figure 5c,d shows the low‐magnification high‐angle annular dark‐field (HAADF) STEM images (so‐called *Z*‐contrast images, *Z*: atomic number) of the Bi_2_S_3_ + 0.25 wt.% FCN sample and densely distributed nanoscale pores can be observed in the matrix grains, consistent with the SEM observations. In order to minimize the influence from STEM sample preparation, we also prepared ground particles (dispersed on holy carbon grids), which exhibit the same nano‐porous structure, as shown in Figure S9. Statistical measurement reveals that the average size of pores is about 30 nm (Figure S9). The EDS result from the area in Figure 5d (see Figure S10) shows that the Fe, Co and Ni are distributed uniformly in the matrix (i.e., FCN incorporated Bi_2_S_3_), which is consistent with the EMPA result.

Two areas marked by the dashed frames are further magnified as Figure 5e,f. In Figure 5e, atomic resolution is obtained along the *c*‐axis and the atomic details match well with the superimposed Bi_2_S_3_ structure (Bi: purple and S: yellow). Except the contrast difference in this image, no other changes are detected, suggesting that the dark contrast area is indeed a nanopore. In Figure 5f, a grain boundary triple junction is revealed. It is seen that the boundaries maintain atomic‐layer contacts and no amorphous regions are present.

Nanosized Bi metals are also found inside the grains (Figure 5g), which show an orientation relationship of <2 $\bar{1}\bar{1}$0>_Bi_ || [001]_Bi2S3_ with the matrix (Figure 5h,i; Figure S11). They usually coexist with nanopores, as shown in Figure 5g; Figure S9 and Figure S11, indicating that the formation of nanosized Bi and nanopores is actually correlated. The most likely mechanism is discussed as follows (see also Figure 1a for demonstration): First, during SPS, small/dispersed FCN particles react with matrix and FCN incorporation to the Bi_2_S_3_ lattice (including replacement of Bi and interstitial site filling) therefore occurs. This leads to the lattice expansion of the FCN incorporated Bi_2_S_3_, as supported by Figure 2, Figure 4 and Figure S10. Second, as a result of replacement, metallic Bi is reduced from the lattice and forms nanosized particles. Since SPS is in a HT, HP and vacuum environment, most of the metallic Bi nanoparticles volatilized (due to the size effect, the smaller the size, the lower the evaporation temperature [52, 53, 54]), leaving densely distributed nanopores together with residual Bi nanoparticles in the grain matrix (e.g., Figure 5g).

**FIGURE 6:**
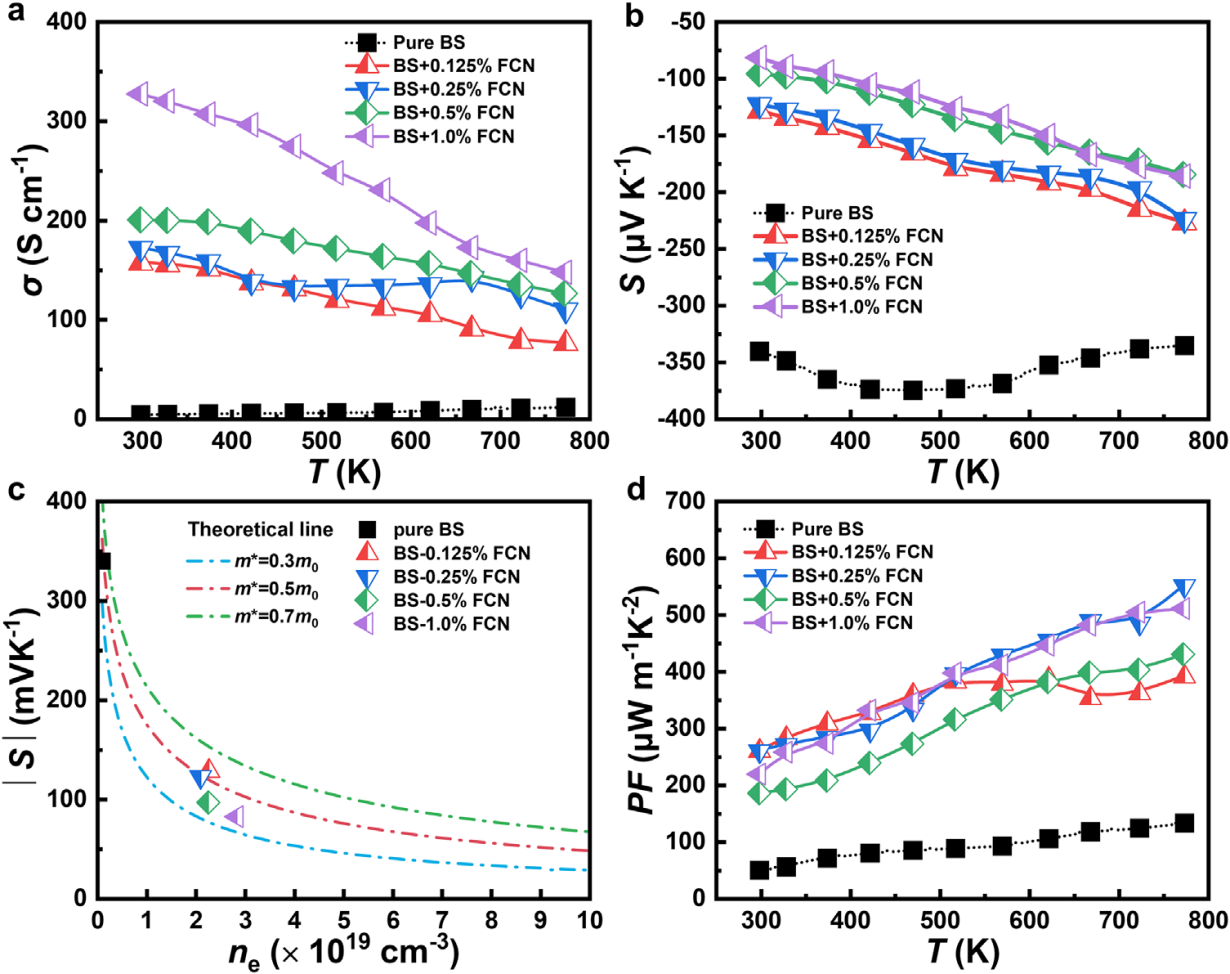
Electrical transport properties of Bi_2_S_3_ + *x* wt.% FCN (*x* = 0, 0.125, 0.25, 0.5, 1.0). (a) *σ*; (b) *S*; (c) Pisarenko curves and (d) *PF*. BS represents Bi_2_S_3_ for short.

For the Bi_2_S_3_ + *x* wt.% FCN bulk samples, *σ* as a function of *T* is shown in Figure 6a. Compared with pure Bi_2_S_3_, the *σ* value is significantly higher for the Bi_2_S_3_ + *x* wt.% FCN samples over the full temperature range. As *x* increases, *σ* increases continuously. At 300 K, it increases from 4 S cm^−1^ for the pure Bi_2_S_3_ sample to 327 S cm^−1^ for the *x* = 1.0 sample, an increase of two orders of magnitude. In addition, *σ* of the FCN treated samples decreases gradually with the increased *T*, indicating that the conductive characteristics changes from that of a semiconductor to a degenerate semiconductor. All of these results indicate that (at least part of) FCN enters the Bi_2_S_3_ lattice and achieves effective doping. This is consistent with the XPS and EDS results.

**FIGURE 7:**
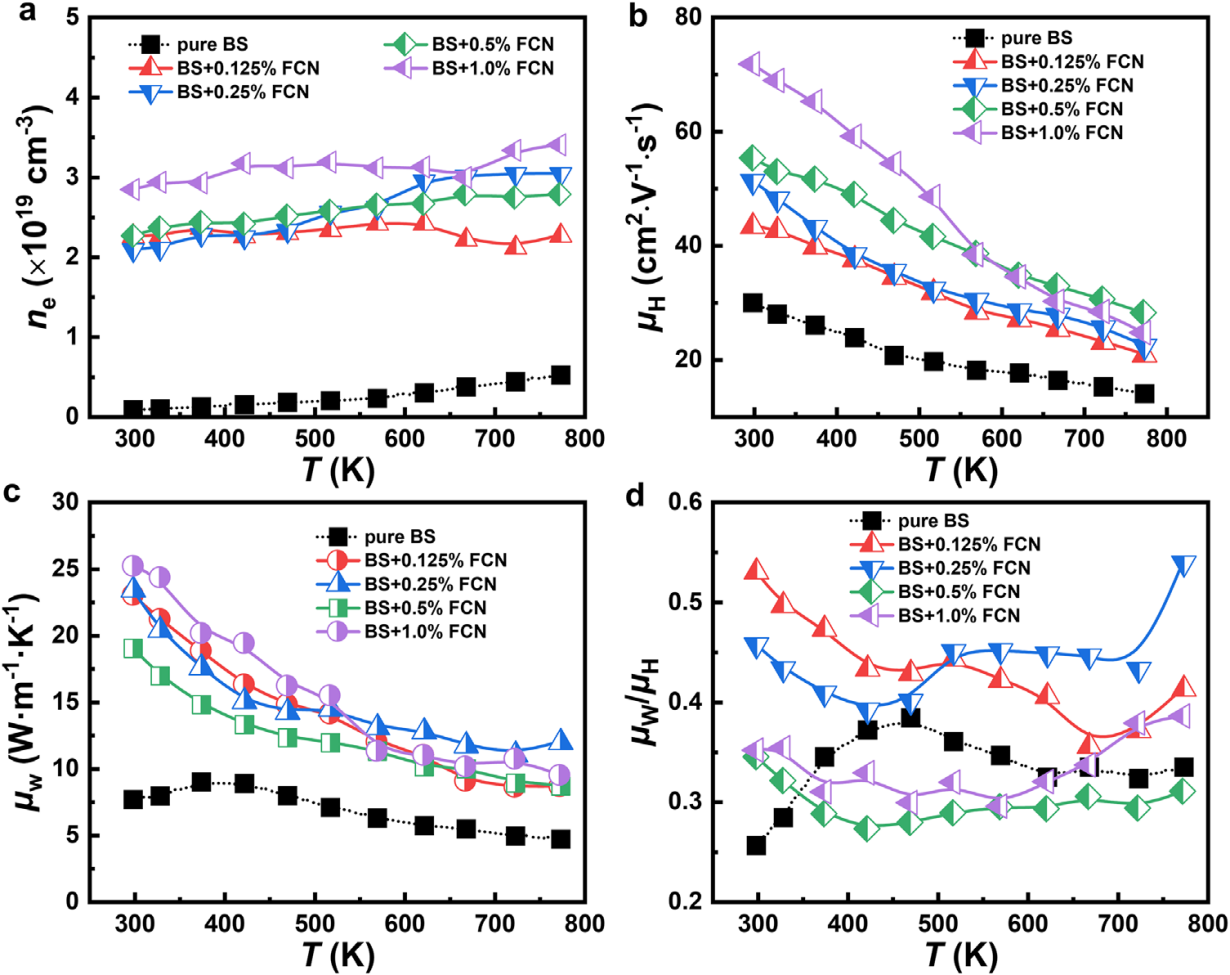
Temperature‐dependent (a) carrier concentration; (b) Hall carrier mobility; (c) weighted mobility, and (d) the ratio of weighted mobility to carrier mobility for Bi_2_S_3_‐*x* wt.% FCN (*x* = 0, 0.125, 0.25, 0.5, 1.0) samples. BS represents Bi_2_S_3_ for short.

The plots of *S* versus *T* for the Bi_2_S_3_ + *x* wt.% FCN samples are shown in Figure 6b. All of the samples show negative *S* values, indicating that they are n‐type semiconductors. Thus, electrons are the main carriers. Opposite to the *σ* case, the *S* values decrease as *x* increases. Figure 6c plots the Pisarenko curves, which demonstrate the relationship between carrier concentration *n*_e_ (subscript e denotes electrons), *S* and the effective mass of the carriers (m*) [55]. It is evident that at 300 K, m* decreases gradually as *x* increases. In combination with the result in Figure 7a, it is found that the decrease in |*S*| as *x* increases is a combined effect from increased *n*_e_ and decreased m*. Particularly, the |*S*| value of the *x* = 0.25 sample increases from 128 at 300 K to 224 µV K^−1^ at 773 K, meaning that a good *S* value is maintained over the measured temperature range. Figure 6d plots the *PF* values as a function of *T*, which will be discussed later.

Furthermore, the effect of FCN on the electrical transport properties is studied using variable‐temperature Hall measurements. Figure 7a plots *n*_e_ as a function of *T* for the Bi_2_S_3_ + *x* wt.% FCN (*x* = 0, 0.125, 0.25, 0.5, and 1.0) samples. The *n*_e_ value of pure Bi_2_S_3_ increases with *T*, primarily due to the thermal excitation of carriers. The FCN incorporated samples have *n*_e_ with negligible *T*‐dependence. Additionally, *n*_e_ increases with *x* due to the enhanced incorporation of FCN to the Bi_2_S_3_ matrix during sintering. The *n*_e_ values are 9.05 × 10^17^ and 2.84 × 10^19^ cm^−3^ for pure Bi_2_S_3_ and the Bi_2_S_3_ + 1.0 wt.% FCN sample, respectively. Figure 7b shows the variation of the carrier mobility *µ*_H_ as a function of *T* for the Bi_2_S_3_ + *x* wt.% FCN (*x* = 0, 0.125, 0.25, 0.5, and 1.0) samples. For all of the samples, *µ*_H_ decreases with increased *T* and the decreasing trend matches the *T* ^−3/2^ law, indicating that the scattering mechanism is primarily phonon scattering. Meanwhile, the *µ*_H_ values increases significantly as *x* increases. At RT, the *µ*_H_ values for the *x* = 0.125 and 1.0 samples are 43 and 72 cm^2^ V^−1^ s^−1^, respectively. Such a significant increase in the carrier mobility can be understood from the following aspects: First, the reduction of Bi_2_S_3_ by FCN during sintering creates metallic Bi, which can act as a grain lubricant and sintering aid. This not only promotes the alignment of nanorods under pressure, but more importantly decreases the stacking induced pores that sever macroscopic conduction paths. Although the newly formed nanopores act as scattering centers, their detrimental impact is far outweighed by the repair of conductive paths resulting from elimination of stacking induced pores. Second, the precipitated Bi metal serves as a bridge for the fast migration of carriers. Third, the FCN‐S phases as a result of FCN and Bi_2_S_3_ reaction usually have excellent *σ*, which have been widely used in energy conversion and storage (e.g., electrocatalysis [56, 57] and supercapacitors [58, 59]).

Now the weighted mobility (*µ*_W_) is calculated using the following formula to better evaluate the intrinsic electrical transport properties of the Bi_2_S_3_ + *x* wt.% FCN samples [60]:

$$\begin{array}{cc} \mu_{W} & =\frac{3h^{3}\sigma }{8\pi {\left(2m_{e}kBT\right)}^{\frac{3}{2}}}\;\\ & \;\times \left[\frac{exp\left[\frac{\left|S\right|}{kB/e}-2\right]}{1+exp\left[-5\left(\frac{\left|S\right|}{kB/e}-1\right)\right]}+\frac{\frac{3}{\pi^{2}}\frac{\left|S\right|}{kB/e}}{1+exp\left[5\left(\frac{\left|S\right|}{kB/e}-1\right)\right]}\right] \end{array}$$

where, *k*_B_, *h*, *m*_e_ and *e* represent the Boltzmann constant, Planck constant, electron mass, and electron charge, respectively. As demonstrated in Figure 7c, the FCN incorporated samples show enhanced *µ*_W_ values than pure Bi_2_S_3_, manifesting improved electrical transport properties. In addition, the ratio between *µ*_W_ and *µ*_H_ (i.e., *µ*_W_/*µ*_H_) (Figure 7d) could study the change in m* according to the following formula [55]:

$$\mu_{W}\approx \mu_{H}{\left(\frac{m^{*}}{m_{e}}\right)}^{\frac{3}{2}}$$

At RT, *µ*_W_/*µ*_H_ decreases as *x* increases, which is consistent with the change in m*, as demonstrated in Figure 6c. After 600 K, the *x* = 1.0 sample shows a significantly increased *µ*_W_/*µ*_H_ value than the *x* = 0.5 sample. This explains why the former has higher *S* value than that of the latter after 600 K.

Now we return to Figure 6d, the FCN incorporated samples exhibit significantly enhanced *PF* than pure Bi_2_S_3_. The Bi_2_S_3_ + 0.25 wt.% FCN sample has the highest *PF* of 551 µW m^−1^ K^−2^ at 773 K. FCN not only optimizes *n*_e_ but also increase *µ*_H_. The increased *σ* coupled with a large *S* value significantly enhances *PF* over a wide temperature range, suggesting that FCN can improve the electrical transport properties of polycrystalline Bi_2_S_3_ over a wide temperature range, thus resulting in a high average *ZT* value. Moreover, repeatability test of *σ* and *S* (Figure S12) and the vacuum annealing results (Figure S13) demonstrate excellent thermal stability of the material.

**FIGURE 8:**
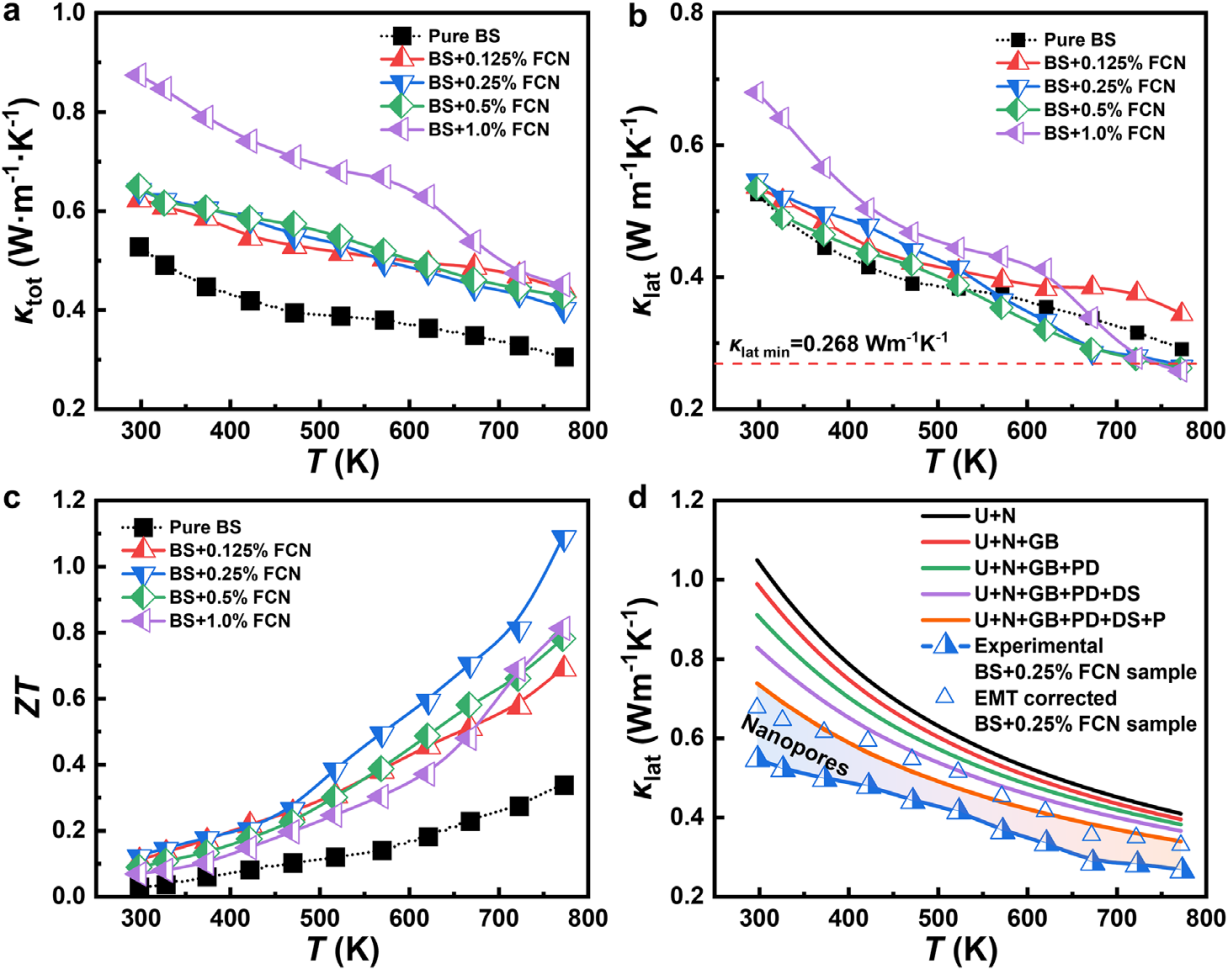
Thermal transport properties of Bi_2_S_3_‐*x* wt.% FCN (*x* = 0, 0.125, 0.25, 0.5, 1.0). (a) Total thermal conductivity, (b) lattice thermal conductivity and (c) *ZT* values. (d) Contribution of different phonon mechanisms calculated by the Debye‐Callaway theory showing the influence of nanopores on *κ*_lat_ of the Bi_2_S_3_ + 0.25 wt.% FCN sample (Cf. text for details). BS represents Bi_2_S_3_ for short.

The thermal transport properties of Bi_2_S_3_ + *x* wt.% FCN (*x* = 0, 0.125, 0.25, 0.5, and 1.0) samples are studied in Figure 8. In Figure 8a, the *κ*_tot_ of the samples increases with the FCN content, suggesting a deteriorated contribution in the thermoelectric performance. The *κ*_tot_ values of pure Bi_2_S_3_ and the *x* = 1.0 sample are 0.52 and 0.87 W m^−1^ K^−1^ at RT, respectively. Nevertheless, considering the great improvement in the electrical properties, such deterioration in thermal transport is mild. Note that *κ*_tot_ consists of two components: *κ*_lat_ and electronic thermal conductivity (*κ*_ele_). The latter can be calculated using *κ*_ele_ = *LσT*, where *L* represents the Lorentz number and can be calculated by assuming the single parabolic band model (SPB) and by fitting the measured *S* values [61]. Specifically, acoustic phonon scattering is assumed as the dominant scattering mechanism (scattering factor *r* = −1/2) for the SPB model calculations, which is supported by the observed *µ*_H_ ∝ *T* ^−3/2^ relationship shown in Figure 7b. Plots of *κ*_lat_ (= *κ*_tot_ – *κ*_ele_) and *κ*_ele_ are presented in Figure 8b and Figure S14, respectively, as a function of *T* for all samples. This allows the individual contribution from *κ*_lat_ and *κ*_ele_ to be evaluated. It is evidenced that the increase in *κ*_tot_ is mainly from the *κ*_ele_ contribution. This is predictable as the increased *n*_e_ caused by FCN incorporation inevitably increases *κ*_ele_. In contrast, the rise of *κ*_lat_ of the FCN treated samples is not significant. Notably, *κ*_lat_ becomes even lower than the pure case when *T* > 550 K for the *x* = 0.25 and 0.5 samples. At 773 K, the values are 0.27 and 0.26 W m^−1^ K^−1^, respectively, reaching the amorphous limiting value of ∼0.27 W m^−1^ K^−1^ for Bi_2_S_3_ based on the Cahill model [62, 63]. By combining results in Figure 6d and Figure 8a, the *ZT* values are plotted in Figure 8c. It is seen that the electrical and thermal properties reach the best compromise for the *x* = 0.25 sample across the entire temperature range and a peak *ZT* of 1.1 is achieved at 773 K, which is almost three times that of pure Bi_2_S_3_.

From the microstructure results, the following issues can be discussed to understand the variation of *κ*_lat_. First, metallic Bi has a high thermal conductivity, thereby, the residual Bi in the FCN treated samples should increase *κ*_lat_ with reference to the pure sample at low temperatures. When the temperature exceeds 544.3 K (i.e., melting point of Bi), the residual Bi becomes liquid, thus decreasing *κ*_lat_ at high temperatures. Second, the reduced size of stacking related pores and the improved grain connectivity in the FCN treated samples should increase *κ*_lat_. Third, densely distributed nanopores are reported to be effective source for phonon scattering. In this regard, their presence should decrease *κ*_lat_ over a wide temperature range.

To further understand the phonon scattering mechanisms, Debye–Callaway model is used to analyze *κ*_lat_ of the Bi_2_S_3_ + 0.25 wt.% FCN sample using the following equation [64, 65]:

$$\kappa_{\mathrm{lat}}=\frac{k_{B}}{2\pi^{2}v}\;{\left(\frac{k_{B}T}{\hbar }\right)}^{3}\int_{0}^{\frac{\theta_{D}}{T}}\tau_{c}\frac{\chi^{4}e^{\chi }}{{\left(e^{\chi }-1\right)}^{2}}d\chi$$

where *ℏ*, *v*, *θ*_D_, *χ* and *τ*_c_ represent Plank's reduced constant, average sound velocity, Debye temperature, reduced phonon frequency (defined as *ℏω*/*k*_B_ *T*, where *ω* is the phonon angular frequency) and total phonon relaxation time, respectively. *τ*_c_ can be expressed using the following equation:

$$\frac{1}{\tau_{c}}=\frac{1}{\tau_{U}}+\frac{1}{\tau_{N}}+\frac{1}{\tau_{\mathrm{GB}}}+\frac{1}{\tau_{\mathrm{PD}}}+\frac{1}{\tau_{\mathrm{DS}}}+\frac{1}{\tau_{P}}$$

where, *τ*_U_, *τ*_N_, *τ*_GB_, *τ*_PD_, *τ*_DS_ and *τ*_P_ represent the relaxation times corresponding to the scattering from the phonon‐phonon U‐process (U), phonon‐phonon N‐process (N), grain/phase boundaries (GB), vacancies/alloy elements (point defects, abbreviated as PD), dislocations (DS), and precipitates (P, mainly related to the FCN‐S and remained Bi second phases), respectively. As seen from Figure 8d, the fitted *κ*_lat_ values using the Debye–Callaway model (see Table S3, for details) deviate from the experimental results, even when the influence from the known defects with different scales on phonon relaxation time has been fully considered. It is therefore reasonable to deduce that the mismatch between experimental and fitted values are caused by nanopores. Typically, point defects scatter high‐frequency phonons, while grain boundaries and micropores target low‐frequency ones. However, mid‐frequency phonons often lack effective scattering mechanisms. The appropriate size of nanopores effectively bridges this gap in the phonon scattering spectrum. Consequently, this multi‐scale defect structure ensures efficient broadband phonon scattering, thereby minimizing the lattice thermal conductivity. Effective medium theory [41] (EMT, *κ*_L,d_ = *κ*_L,p_/(1 – 3*ε*/2), where *κ*_L,d_, *κ*_L,p_ represent the lattice thermal conductivity of dense and porous materials and *ε* denotes porosity, respectively) is applied to obtain the corrected *κ*_L,d_ of the dense Bi_2_S_3_ + 0.25 wt.% FCN sample. It can be seen that the values after EMT correction are close to the fitted ones, demonstrating that the nanoporous structures do have an optimizing effect (i.e., ∼20% reduction) on *κ*_lat_. Similar findings have also been reported in literature [39, 41, 66].

**FIGURE 9:**
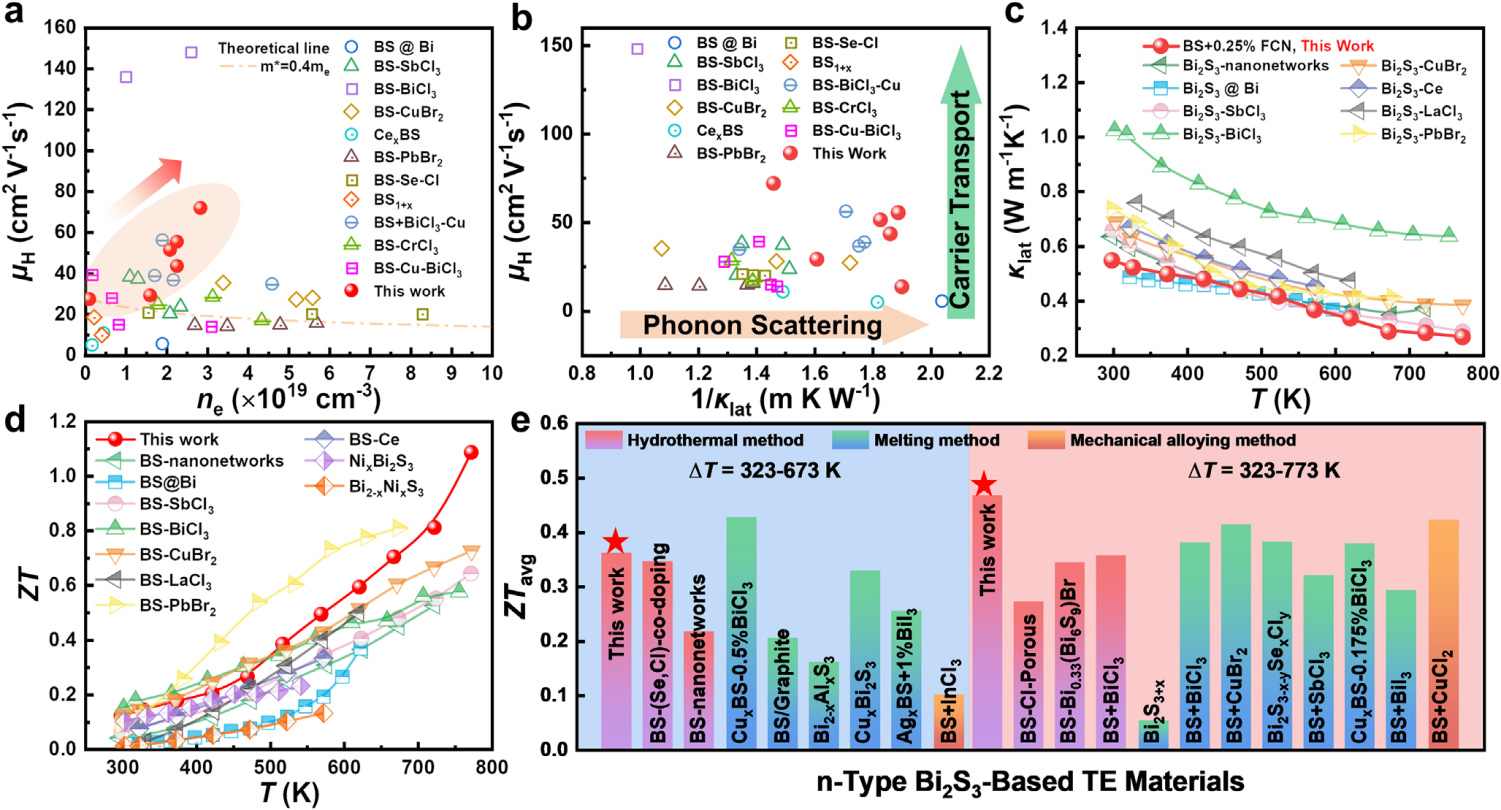
(a) Carrier mobility as a function of carrier density and its comparison with other Bi_2_S_3_‐based samples. (b) Relationship between carrier mobility and the reciprocal of lattice thermal conductivity and its comparison with other Bi_2_S_3_‐based samples. (c) Lattice thermal conductivity of Bi_2_S_3_ + 0.25 wt.% FCN sample in comparison with other Bi_2_S_3_‐based samples. (d) *ZT* of this work in comparison with other Bi_2_S_3_‐based samples and (e) *ZT*_avg_ of this work in comparison with other Bi_2_S_3_‐based samples at 323 – 673 K and 323 – 773 K ranges. Note that all comparative data cited in this figure are based on polycrystalline bulk materials. BS represents Bi_2_S_3_ for short.

Our results are compared with the reported values in literature, some of the key parameters are displayed in Figure 9. Figure 9a shows the RT *µ*_H_ values as a function of *n*_e_. Notably, the FCN introduction strategy implemented in this study yields a *µ*_H_ value superior to those of other doped Bi_2_S_3_‐based materials, such as Bi_2_S_3_ doped with SbCl_3_ [67], CuBr_2_ [68], PbBr_2_ [69], Cu‐0.175 mol% BiCl_3_ [70], 0.5 mol% BiCl_3_‐Cu [71], and others [32, 37, 72, 73, 74]. This observation corroborates the premise that the introduction of FCN could maintain a commendable *µ*_H_ while increasing *n*_e_. Figure 9b summarizes the relationships between *µ*_H_ and 1/*κ*_lat_ for the *x* = 0.25 sample and other excellent Bi_2_S_3_‐based materials at 300 K [32, 37, 67, 68, 69, 70, 71, 72, 73, 74, 75], which highlights the superior balance in electronic and thermal transport properties due to the introduction of FCN in this study. The FCN‐treated sample with values located at the upper right corner exhibits a more desirable combination of high *µ*_H_ and low *κ*_lat_ than other Bi_2_S_3_‐based materials. Figure 9c further compares the *T* dependent *κ*_lat_ values between the *x* = 0.25 sample and other reported systems [32, 37, 38, 67, 68, 69, 75, 76]. It is obvious that our result is among the best. This is benefited from the present engineering strategy that introduces microstructures covering very broad dimensions, including interfaces (∼1 nm), pores (∼30 nm), residual Bi (nm to µm), FCN‐S (several to hundreds of micrometers).

We also compare *ZT*, in particular the peak values [32, 37, 38, 67, 68, 69, 75, 76, 77, 78] (Figure 9d) and the average values (i.e., *ZT*_avg_ [32, 35, 38, 62, 67, 68, 70, 71, 72, 73, 79, 80, 81, 82, 83, 84, 85, 86, 87]) over the temperature range of 323 – 673 and 323 – 773 K (Figure 9e). It can be seen that the widely used strategy by employing metal dopants to Bi_2_S_3_ does not achieve excellent *ZT* value (usually < 1.0). Instead, by introducing FCN treatment, a record‐high peak *ZT* of 1.1 is achieved at 773 K in this work. In addition to the peak *ZT*, the *ZT*_avg_ value can be also improved. Although the value of 0.36 in the 323 – 673 K range is slightly lower than that of the Cu‐BiCl_3_ co‐doped sample [71], the *ZT*_avg_ in the 323 – 773 K range demonstrates the best performance of about 0.47 among the reported Bi_2_S_3_‐based materials.

**FIGURE 10:**
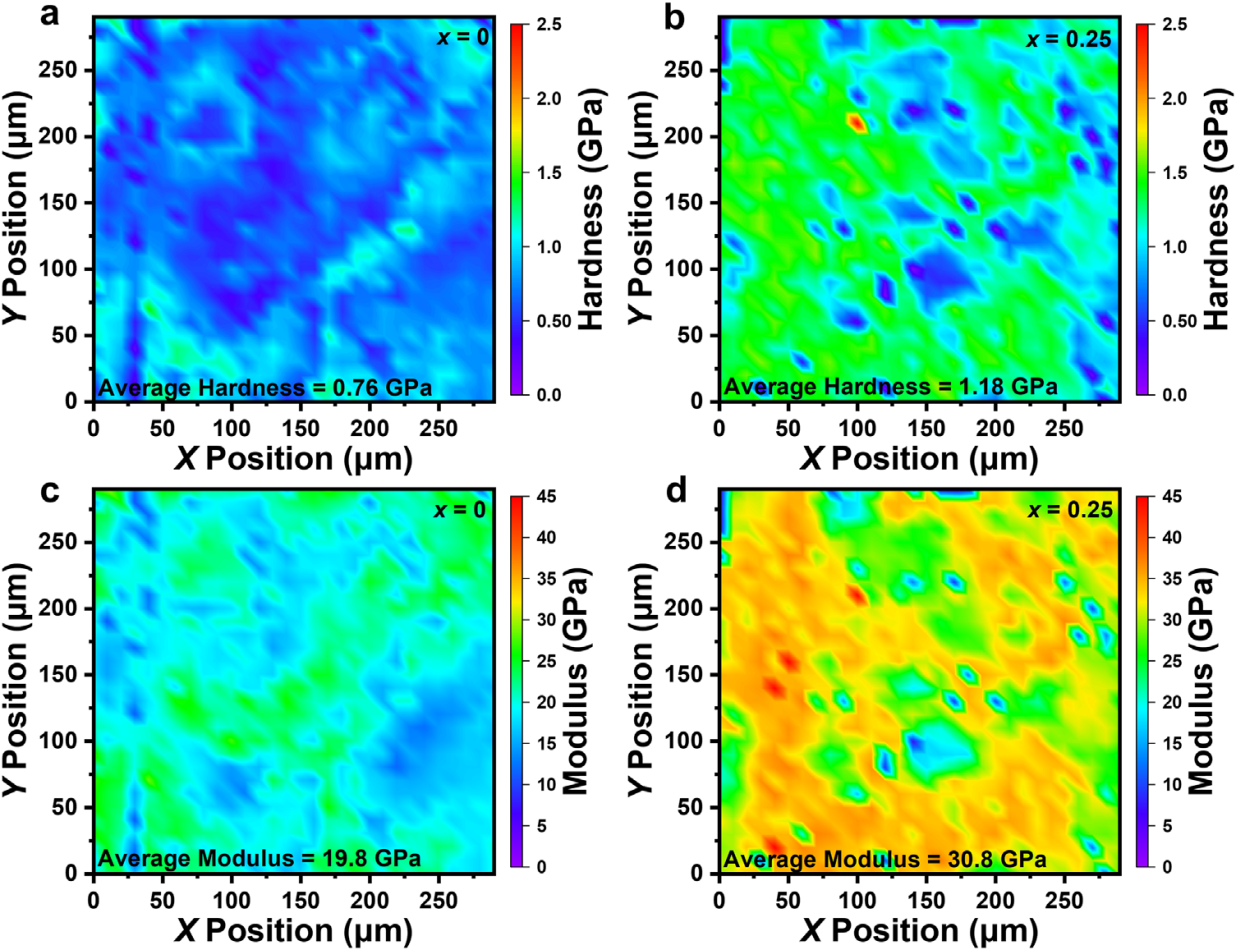
2D contour maps of (a,b) hardness and (c,d) Young's modulus for the pure Bi_2_S_3_ and Bi_2_S_3_ + 0.25 wt.% FCN samples.

The mechanical properties are important for device application. Here, the mechanical properties of the Bi_2_S_3_ + *x* wt.% FCN (*x* = 0, 0.125, 0.25, 0.5, and 1.0) samples are investigated using nanoindentation with the load force set at 20 mN. Figure 10 shows the two‐dimensional (2D) contour plots of the hardness and Young's modulus for the pure and *x* = 0.25 samples. In both samples, the hardness and Young's modulus show position related variations, as evidenced by the color mapping fluctuations. However, the average values for both parameters are significantly improved in the FCN treated sample. Specifically, the hardness increases from 0.76 to 1.18 GPa and the Young's modulus from 19.8 to 30.8 GPa, which are about 55% enhancement. In addition, we find that increasing FCN enhances the mechanical properties, as shown in Figures S15 and S16.

Last but not least, the same strategy has been applied to other chalcogenide thermoelectric systems, including PbS and SnS, and the results are given in Figures S17 and S18, respectively. After adding 1 wt.% FCN, diffraction peaks of Pb are observed in XRD, confirming FCN's capability in reducing Pb. The fracture surface morphology images show increased pore density in the FCN treated sample with reference to the pure PbS. As for SnS, although we don't observe clear Sn diffraction peaks in the SnS + *x* wt.% FCN (*x* = 0, 1) sample, the grains in the treated sample become more rounded in comparison with the pure sample and the gaps between grains also increase. All of the results indicate that FCN can be used as a universal modifier for chalcogenide thermoelectric materials. Further investigations are therefore desirable.

## 3 Conclusion

In conclusion, this study demonstrates a promising strategy that utilizes the difference in chemical activity between metal elements and the volatile nature of Bi to significantly improve the thermoelectric performance of n‐type binary polycrystalline Bi_2_S_3_. Reaction between Bi_2_S_3_ with FCN powders during SPS results in effective incorporation of FCN to Bi_2_S_3_ lattice on the one hand. On the other hand, it leads to the reduction reaction that forms metallic Bi and FCN‐S compounds within the FCN incorporated Bi_2_S_3_ matrix. The FCN incorporation and the conductive precipitate phases formed synergistically enhance *n*_e_ and *µ*_H_. In combination with the significantly increased *σ* and the relatively high *S* values, a highly enhanced *PF* of about 551 µW m^−1^ K^−2^ is obtained at 773 K for the Bi_2_S_3_ + 0.25 wt.% FCN sample. In parallel, the volatility of nanosized Bi during SPS triggers the formation of nanopores, which, in combination with the presence of multiscale lattice defects, contribute to the effective scattering of all‐scale phonons in the whole temperature range and maintain a low *κ*_lat_ of 0.27 W m^−1^ K^−1^ at 773 K for Bi_2_S_3_ + 0.25 wt.% FCN sample. Consequently, an excellent peak *ZT* of 1.1 is realized at 773 K in the n‐type Bi_2_S_3_ + 0.25 wt.% FCN polycrystals, suggesting that Bi_2_S_3_ is a promising thermoelectric material for industrial applications. The strategy used in this study provides a valuable reference for other thermoelectric material systems.

## 4 Experimental Section

The experimental details can be found in the Supporting information.

## Funding

National Natural Science Foundation of China (Grant No. 52562030), National Key R&D Program of China (Grant No. 2022YFF0503804), Academician (Expert) Workstation of Yunnan Province Program (Grant No. 202405AF140066), Yunnan Science and Technology Program (Grant No. 202401AT070403), Outstanding Youth Fund of Yunnan Province (Grant No. 202201AV070005), National Natural Science Foundation of China (Grant No. 52162029) and Yunnan Major Scientific and Technological Projects (Grant No. 202302AG050010).

## Conflicts of Interest

The authors declare no conflict of interest.

## Supporting information




**Supporting File**: adma71998‐sup‐0001‐SuppMat.docx

## Data Availability

The data that support the findings of this study are available from the corresponding author upon reasonable request.;

## References

[1] F. J. DiSalvo , “Thermoelectric Cooling and Power Generation,” Science 285 (1999): 703–706, 10.1126/science.285.5428.703. 10426986

[2] R. Y. Nuwayhid , A. Shihadeh , and N. Ghaddar , “Development and testing of a domestic woodstove thermoelectric generator With natural convection cooling,” Energy Conversion and Management 46 (2005): 1631–1643, 10.1016/j.enconman.2004.07.006.

[3] L. E. Bell , “Cooling, Heating, Generating Power, and Recovering Waste Heat With Thermoelectric Systems,” Science 321 (2008): 1457–1461, 10.1126/science.1158899. 18787160

[4] D. Liu , D. Wang , T. Hong , et al., “Lattice plainification advances highly effective SnSe crystalline thermoelectrics,” Science 380 (2023): 841–846, 10.1126/science.adg7196. 37228203

[5] Y. Qin , B. Qin , T. Hong , et al., “Grid‐plainification enables medium‐temperature PbSe thermoelectrics to cool better Than Bi_2_Te_3_ ,” Science 383 (2024): 1204–1209, 10.1126/science.adk9589. 38484057

[6] H. Liang , J. Guo , Y.‐X. Zhou , Z.‐Y. Wang , J. Feng , and Z.‐H. Ge , “CuPbBi_5_S_9_ thermoelectric material With an intrinsic low thermal conductivity: Synthesis and properties,” Journal of Materiomics 8 (2022): 174–183, 10.1016/j.jmat.2021.03.016.

[7] Y. Jin , Y. Qiu , S. Bai , et al., “Modifying Roles of CuSbSe_2_ in Realizing High Thermoelectric Performance of GeTe,” Advanced Energy Materials 14 (2024): 2400623, 10.1002/aenm.202400623.

[8] H.‐N. Shi , S.‐L. Bai , Y.‐P. Wang , et al., “Contrasting strategies of optimizing carrier concentration in bulk InSe for enhanced thermoelectric performance,” Rare Metals 43 (2024): 4425–4432, 10.1007/s12598-024-02756-z.

[9] S. Zhan , S. Bai , B. Qin , et al., “High Carrier Mobility Promotes In‐Plane Thermoelectric Performance of n‐Type PbSnS 2 Crystals,” Advanced Functional Materials 34 (2024): 2406428, 10.1002/adfm.202406428.

[10] M. Li , X. Zhao , D. Wang , et al., “Enhancing the Thermoelectric and Mechanical Properties of p‐Type PbS Through Band Convergence and Microstructure Regulation,” Nano Letters 24 (2024): 8126–8133, 10.1021/acs.nanolett.4c02058. 38904329

[11] X. Qian , H.‐R. Guo , J.‐X. Lyu , et al., “Enhancing thermoelectric performance of p‐type SnTe Through manipulating energy band structures and decreasing electronic thermal conductivity,” Rare Metals 43 (2024): 3232–3241, 10.1007/s12598-024-02663-3.

[12] B. Qin , D. Wang , X. Liu , et al., “Power generation and thermoelectric cooling enabled by momentum and energy multiband alignments,” Science 373 (2021): 556–561, 10.1126/science.abi8668. 34326238

[13] T. Chen , S. Li , K. Chen , et al., “Enhancing Thermoelectric Performance of n‐Type Bi_2_Te_2.7_Se_0.3_ Through Incorporation of Amorphous Si_3_N_4_ Nanoparticles,” ACS Applied Materials and Interfaces 16 (2024): 22016–22024. 38647228 10.1021/acsami.4c02652

[14] Z. Fan , J. Liang , J.‐L. Chen , et al., “Realizing high thermoelectric performance for p‐type SiGe in medium temperature region via TaC compositing,” Journal of Materiomics 9 (2023): 984–991, 10.1016/j.jmat.2023.03.004.

[15] J. Yu , X. Liu , H. Hu , et al., “Ultralow thermal conductivity and high ZT of Cu_2_Se‐based thermoelectric materials mediated by TiO_2−n_ nanoclusters,” Joule 8 (2024): 2652–2666, 10.1016/j.joule.2024.06.007.

[16] K. Jin , J. Tiwari , T. Feng , Y. Lou , and B. Xu , “Realizing high thermoelectric performance in eco‐friendly Bi_2_S_3_ With nanopores and Cl‐doping Through shape‐controlled nano precursors,” Nano Energy 100 (2022): 107478, 10.1016/j.nanoen.2022.107478.

[17] W. Zhang , B. A. Al‐Maythalony , F. Gao , et al., “Thermally stable inorganic Bi 0.4 Sb 1.6 Te 3 /metal–organic framework (MOF) composites With 1‐by‐1 nm pore engineering towards mid‐temperature thermoelectrics,” Energy & Environmental Science 17 (2024): 5679–5690, 10.1039/D4EE01652A.

[18] Z. Yang , Y. Han , Y. Liang , et al., “Chalcogenide perovskite BaZrS_3_ bulks for thermoelectric conversion With ultra‐high carrier mobility and low thermal conductivity,” Acta Materialia 276 (2024): 120156, 10.1016/j.actamat.2024.120156.

[19] P.‐F. Liu , X. Li , J. Li , et al., “Strong low‐energy rattling modes enabled liquid‐Like ultralow thermal conductivity in a well‐ordered solid,” National Science Review 11 (2024): nwae216, 10.1093/nsr/nwae216. 39554237 PMC11562843

[20] K. Guo , J. Zhang , X. Yu , et al., “In‐Plane Overdamping and Out‐Plane Localized Vibration Contribute to Ultralow Lattice Thermal Conductivity of Zintl Phase KCdSb,” Advanced Science 11 (2024): 2402209, 10.1002/advs.202402209. 38946664 PMC11633356

[21] F. Hao , P. Qiu , Y. Tang , et al., “High efficiency Bi_2_Te_3_‐based materials and devices for thermoelectric power generation Between 100 and 300°C,” Energy & Environmental Science 9 (2016): 3120–3127, 10.1039/C6EE02017H.

[22] B. Zhu , X. Liu , Q. Wang , et al., “Realizing record high performance in n‐type Bi_2_Te_3_‐based thermoelectric materials,” Energy & Environmental Science 13 (2020): 2106–2114, 10.1039/D0EE01349H.

[23] Y.‐K. Zhu , Y. Sun , X. Dong , et al., “General design of high‐performance and textured layered thermoelectric materials via stacking of mechanically exfoliated crystals,” Joule 8 (2024): 2412–2424, 10.1016/j.joule.2024.05.006.

[24] H.‐L. Zhuang , H. Hu , J. Pei , et al., “High ZT in p‐Type Thermoelectric (Bi,Sb)2Te3 with Built‐in Nanopores,” Energy and Environmental Science 15 (2022): 2039–2048.

[25] Y. Xiao , H. Wu , W. Li , et al., “Remarkable Roles of Cu To Synergistically Optimize Phonon and Carrier Transport in n‐Type PbTe‐Cu_2_Te,” Journal of the American Chemical Society 139 (2017): 18732–18738, 10.1021/jacs.7b11662. 29182275

[26] H. J. Wu , L. D. Zhao , F. S. Zheng , et al., “Broad temperature plateau for thermoelectric figure of merit ZT>2 in phase‐separated PbTe0.7S0.3,” Nature Communications 5 (2014): 4515. 10.1038/ncomms5515 25072798

[27] B. Jiang , Y. Yu , J. Cui , et al., “High‐entropy‐stabilized chalcogenides With high thermoelectric performance,” Science 371 (2021): 830–834, 10.1126/science.abe1292. 33602853

[28] S. Cai , S. Hao , Z.‐Z. Luo , et al., “Discordant nature of Cd in PbSe: Off‐centering and core–shell nanoscale CdSe precipitates lead to high thermoelectric performance,” Energy & Environmental Science 13 (2020): 200–211, 10.1039/C9EE03087E.

[29] W. Li , L. Zheng , B. Ge , et al., “Promoting SnTe as an Eco‐Friendly Solution for p‐PbTe Thermoelectric via Band Convergence and Interstitial Defects,” Advanced Materials 29 (2017): 1605887, 10.1002/adma.201605887. 28247491

[30] Z. Li , X. He , H. Li , C. Wang , Y. Niu , and J. Jiang , “The effect of thermoelectric augmentation dramatically increased the specific capacity for electrochemical energy storage,” Chemical Engineering Journal 495 (2024): 153535, 10.1016/j.cej.2024.153535.

[31] J. Chen , Z. Dong , Q. Li , et al., “Enhanced Thermoelectric Performance in Vacancy‐Filling Heuslers due to Kondo‐Like Effect,” Advanced Materials 36 (2024): 2405858, 10.1002/adma.202405858. 38899584

[32] K. Biswas , L. D. Zhao , M. G. Kanatzidis , and T.‐F. Thermoelectric , “The Anisotropic n‐Type Semiconductor Bi2S3,” Advanced Energy Materials 2 (2012): 634–638.

[33] Z.‐H. Ge , B.‐P. Zhang , Y.‐Q. Yu , and P.‐P. Shang , “Fabrication and properties of Bi_2−x_Ag_3x_S_3_ thermoelectric polycrystals,” Journal of Alloys and Compounds 514 (2012): 205–209, 10.1016/j.jallcom.2011.11.072.

[34] J. Yang , H. Ye , X. Zhang , et al., “Dual‐Site Doping and Low‐Angle Grain Boundaries Lead to High Thermoelectric Performance in N‐Type Bi 2 S 3,” Advanced Functional Materials 34 (2024): 2306961, 10.1002/adfm.202306961.

[35] J. Yang , L. X. Yu , T. T. Wang , et al., “Thermoelectric properties of n‐type Cu Bi2S3 materials fabricated by plasma activated sintering,” Journal of Alloys and Compounds 780 (2019): 35–40, 10.1016/j.jallcom.2018.11.343.

[36] H. Liu , L. Zhang , Y. Shen , et al., “Enhanced thermoelectric performance of Bi_2‐x_Cu_x_S_3_ by hydrothermal synthesis and spark plasma sintering,” Ceramics International 49 (2023): 36130–36136, 10.1016/j.ceramint.2023.08.292.

[37] J. Pei , L. J. Zhang , B. P. Zhang , P. P. Shang , and Y. C. Liu , “Enhancing the thermoelectric performance of Ce_x_Bi_2_S_3_ by optimizing the carrier concentration combined With band engineering,” Journal of Materials Chemistry C 5 (2017): 12492–12499, 10.1039/C7TC04082B.

[38] W. Liu , C. F. Guo , M. Yao , et al., “Bi2S3 nanonetwork as precursor for improved thermoelectric performance,” Nano Energy 4 (2014): 113–122, 10.1016/j.nanoen.2013.12.015.

[39] B. Xu , T. Feng , M. T. Agne , et al., “Highly Porous Thermoelectric Nanocomposites With Low Thermal Conductivity and High Figure of Merit From Large‐Scale Solution‐Synthesized Bi 2 Te 2.5 Se 0.5 Hollow Nanostructures,” Angewandte Chemie International Edition 56 (2017): 3546–3551, 10.1002/anie.201612041. 28079961

[40] H. Ju , K. Kim , D. Park , and J. Kim , “Fabrication of porous SnSeS nanosheets With controlled porosity and their enhanced thermoelectric performance,” Chemical Engineering Journal 335 (2018): 560–566.

[41] H. Hu , H.‐L. Zhuang , Y. Jiang , et al., “Thermoelectric Cu 12 Sb 4 S 13 ‐Based Synthetic Minerals With a Sublimation‐Derived Porous Network,” Advanced Materials 33 (2021): 2103633, 10.1002/adma.202103633. 34494316

[42] K. P. Zhao , H. Z. Duan , N. Raghavendra , et al., “Solid‐State Explosive Reaction for Nanoporous Bulk Thermoelectric Materials,” Advanced Materials 29 (2017): 1701148. 10.1002/adma.201701148 28961340

[43] C. B. Tang , Y. Liu , L. G. Ye , Y. M. Chen , M. T. Tang , and S. H. Yang , “Production of bismuth by directly reducing‐matting smelting From bismuth sulfide concentrate,” Chin J Nonferrous Metals 27 (2017): 363–370.

[44] Z.‐H. Ge , B.‐P. Zhang , Z.‐X. Yu , and J.‐F. Li , “Effect of spark plasma sintering temperature on thermoelectric properties of Bi_2_S_3_ polycrystal,” Journal of Materials Research 26 (2011): 2711–2718, 10.1557/jmr.2011.273.

[45] R. B. Shalvoy , G. B. Fisher , and P. J. Stiles , “Bond ionicity and structural stability of some average‐valence‐five materials studied by x‐ray photoemission,” Physical Review B 15 (1977): 1680–1697, 10.1103/PhysRevB.15.1680.

[46] J. Guo , Z. Ge , M. Hu , P. Qin , and J. Feng , “Facile Synthesis of NaBiS_2_ Nanoribbons as a Promising Visible Light‐Driven Photocatalyst,” Physica status solidi (RRL) 12 (2018): 1800135.

[47] J. Guo , Q. Lou , Y. Qiu , et al., “Remarkably enhanced thermoelectric properties of Bi_2_S_3_ nanocomposites via modulation doping and grain boundary engineering,” Applied Surface Science 520 (2020): 146341.

[48] M. Guo , L. Zhou , Y. Li , Q. Zheng , F. Xie , and D. Lin , “Unique nanosheet–nanowire structured CoMnFe layered triple hydroxide arrays as self‐supporting electrodes for a high‐efficiency oxygen evolution reaction,” Journal of Materials Chemistry A 7 (2019): 13130–13141, 10.1039/C9TA01531K.

[49] Y.‐G. Feng , H.‐J. Niu , L.‐P. Mei , J.‐J. Feng , K.‐M. Fang , and A.‐J. Wang , “Engineering 3D hierarchical thorn‐Like PtPdNiCu alloyed nanotripods With enhanced performances for methanol and ethanol electrooxidation,” Journal of Colloid and Interface Science 575 (2020): 425–432, 10.1016/j.jcis.2020.04.120. 32402824

[50] J. Hao , Z. Zhuang , K. Cao , et al., “Unraveling the electronegativity‐dominated intermediate adsorption on high‐entropy alloy electrocatalysts,” Nature Communications 13 (2022): 2662, 10.1038/s41467-022-30379-4. PMC9106752 35562523

[51] P. Brož , J. Vřešťál , J. Sopoušek , et al., “High entropy alloys (FeCoNi)_0.75_Cr_0.25‐x_Cu_x_ – thermal stability and physical properties,” J Alloy Compd 993 (2024): 174628.

[52] S. Xiong , W. Qi , Y. Cheng , B. Huang , M. Wang , and Y. Li , “Universal relation for size dependent thermodynamic properties of metallic nanoparticles,” Physical Chemistry Chemical Physics 13 (2011): 10652–10660, 10.1039/c0cp90161j. 21523307

[53] A. Eremin and E. Gurentsov , “Evaporation temperature depression With decrease of iron nanoparticle size. Validation of semi‐empirical models,” Materials Chemistry and Physics 228 (2019): 180–186, 10.1016/j.matchemphys.2019.02.052.

[54] T. H. Wang , Y. F. Zhu , and Q. Jiang , “Size effect on evaporation temperature of nanocrystals,” Materials Chemistry and Physics 111 (2008): 293–295, 10.1016/j.matchemphys.2008.04.010.

[55] J. Zhu , X. Zhang , M. Guo , et al., “Restructured single parabolic band model for quick analysis in thermoelectricity,” Npj Computational Materials 7 (2021): 116, 10.1038/s41524-021-00587-5.

[56] Y. Wen , Y. Liu , T. Wang , et al., “High‐Mass‐Loading Ni–Co–S Electrodes With Unfading Electrochemical Performance for Supercapacitors,” ACS Applied Energy Materials 4 (2021): 6531–6541, 10.1021/acsaem.1c00557.

[57] J. Hu , Y. Shi , L. Sun , et al., “MOF‐derived spherical Ni S /carbon With B‐doping enabling high supercapacitive performance,” Journal of Materials Science & Technology 153 (2023): 219–227, 10.1016/j.jmst.2022.11.065.

[58] X. Ren , Y. Zhou , Y. Du , et al., “Facile ion exchange to construct Ni‐Fe‐Co sulfides and hydroxides ultrathin nanosheets With rich interfaces for advanced all‐solid‐state asymmetric supercapacitors,” Applied Surface Science 514 (2020): 145951, 10.1016/j.apsusc.2020.145951.

[59] Y. Gao , X. Yue , Y. Dong , Q. Zheng , and D. Lin , “High‐efficiency activated phosphorus‐doped Ni_2_S_3_/Co_3_S_4_/ZnS nanowire/nanosheet arrays for energy storage of supercapacitors,” Journal of Colloid and Interface Science 658 (2024): 441–449, 10.1016/j.jcis.2023.12.099. 38118190

[60] G. J. Snyder , A. H. Snyder , M. Wood , et al., “Weighted Mobility,” Advanced Materials 32 (2020): 2001537, 10.1002/adma.202001537. 32410214

[61] H. S. Kim , Z. M. Gibbs , Y. L. Tang , H. Wang , and G. J. Snyder , “Characterization of Lorenz number With Seebeck coefficient measurement,” APL Materials 3 (2015): 041506, 10.1063/1.4908244.

[62] J. Guo , J. Yang , Z.‐H. Ge , et al., “Realizing High Thermoelectric Performance in Earth‐Abundant Bi 2 S 3 Bulk Materials via Halogen Acid Modulation,” Advanced Functional Materials 31 (2021): 2102838, 10.1002/adfm.202102838.

[63] D. G. Cahill , S. K. Watson , and R. O. Pohl , “Lower limit to the thermal conductivity of disordered crystals,” Physical Review B 46 (1992): 6131–6140, 10.1103/PhysRevB.46.6131. 10002297

[64] J. Callaway and H. C. von Baeyer , “Effect of Point Imperfections on Lattice Thermal Conductivity,” Physical Review 120 (1960): 1149–1154, 10.1103/PhysRev.120.1149.

[65] R. Li , G. Wu , Q. Zhang , et al., “Robust (Bi,Sb)_2_Te_3_ Thermoelectrics Due to Engineered Ion Confinement and Microstructure for Advancing Thermoelectric Power Generators,” Advanced Functional Materials 35 (2025): 2502535, 10.1002/adfm.202502535.

[66] A. U. Khan , K. Kobayashi , D.‐M. Tang , et al., “Nano‐micro‐porous skutterudites With 100% enhancement in ZT for high performance thermoelectricity,” Nano Energy 31 (2017): 152–159, 10.1016/j.nanoen.2016.11.016.

[67] J. Yang , J. N. Yan , G. W. Liu , Z. Q. Shi , and G. J. Qiao , “Improved thermoelectric properties of n‐type Bi_2_S_3_ via grain boundaries and in‐situ nanoprecipitates,” Journal of the European Ceramic Society 39 (2019): 1214–1221, 10.1016/j.jeurceramsoc.2018.11.053.

[68] Z. H. Liu , Y. L. Pei , H. Y. Geng , et al., “Enhanced thermoelectric performance of Bi_2_S_3_ by synergistical action of bromine substitution and copper nanoparticles,” Nano Energy 13 (2015): 554–562, 10.1016/j.nanoen.2015.03.036.

[69] J. Guo , Y.‐X. Zhang , Z.‐Y. Wang , et al., “High thermoelectric properties realized in earth‐abundant Bi_2_S_3_ bulk via carrier modulation and multi‐nano‐precipitates synergy,” Nano Energy 78 (2020): 105227, 10.1016/j.nanoen.2020.105227.

[70] J. Guo , Z.‐Y. Wang , Y.‐K. Zhu , L. Chen , J. Feng , and Z.‐H. Ge , “Synergistically enhanced thermoelectric properties of Bi_2_S_3_ bulk materials via Cu interstitial doping and BiCl_3_ alloying,” Rare Metals 41 (2022): 931–941, 10.1007/s12598-021-01848-4.

[71] Y. Zhu , X. Wang , Y. Shen , Y. Xu , F. Du , and J. Yang , “High thermoelectric performance in n‐type bismuth sulfide by carrier concentration tuning and dense nanodomains,” Journal of the European Ceramic Society 44 (2024): 5096–5104, 10.1016/j.jeurceramsoc.2024.02.025.

[72] Y. Chen , D. Y. Wang , Y. L. Zhou , et al., “Enhancing the thermoelectric performance of Bi_2_S_3_: A promising earth‐abundant thermoelectric material,” Frontiers of Physics 14 (2019): 013601, 10.1007/s11467-018-0845-4.

[73] Y. Wu , J. Pei , R. Zhang , Z. C. Huang , Z. Zhao , and B. P. Zhang , “Temperature gradient cooling technique: Boosting high power factor of Bi2S3+ thermoelectric material,” Journal of Alloys and Compounds 830 (2020): 154451, 10.1016/j.jallcom.2020.154451.

[74] R. Fortulan , S. Aminorroaya Yamini , C. Nwanebu , et al., “Thermoelectric Performance of n‐Type Magnetic Element Doped Bi_2_S_3_ ,” ACS Applied Energy Materials 5 (2022): 3845–3853, 10.1021/acsaem.2c00295. 35573054 PMC9096796

[75] Z. H. Ge , P. Qin , D. S. He , et al., “Highly Enhanced Thermoelectric Properties of Bi/Bi2S3 Nanocomposites,” ACS Applied Materials and Interfaces 9 (2017): 4828–4834. 28084071 10.1021/acsami.6b14803

[76] Y. Wu , Q. Lou , Y. Qiu , et al., “Highly enhanced thermoelectric properties of nanostructured Bi_2_S_3_ bulk materials via carrier modification and multi‐scale phonon scattering,” Inorganic Chemistry Frontiers 6 (2019): 1374–1381, 10.1039/C9QI00213H.

[77] F. Fitriani , S. M. Said , S. Rozali , et al., “Enhancement of Thermoelectric Properties in Cold Pressed Nickel Doped Bismuth Sulfide Compounds,” Electronic Materials Letters 14 (2018): 689–699, 10.1007/s13391-018-0072-8.

[78] W. Ji , H. Yu , X. Li , et al., “Influence of Ni doping on the thermoelectric properties of Bi_2_S_3_ via high pressure and high temperature,” Journal of Alloys and Compounds 966 (2023): 171575, 10.1016/j.jallcom.2023.171575.

[79] H. Hou , J. Yang , G. Liu , X. Zhang , and G. Qiao , “Effects of cation doping on thermoelectric properties of Bi_2_S_3_ materials,” Journal of Materials Science: Materials in Electronics 33 (2022): 22291–22299.

[80] J. Guo , Z. H. Ge , F. Qian , D. H. Lu , and J. Feng , “Achieving high thermoelectric properties of Bi_2_S_3_ via InCl_3_ doping,” Journal of Materials Science 55 (2020): 263–273, 10.1007/s10853-019-04008-3.

[81] F. Anjum , P. Dixit , and T. Maiti , “Enhanced thermoelectric performance With improved mechanical strength in Bi_2_S_3_/graphite composites,” Carbon 218 (2024): 118692, 10.1016/j.carbon.2023.118692.

[82] J. N. Yan , J. Yang , B. Z. Ge , et al., “Effect of Silver and Iodine Co‐doping on the Thermoelectric Properties of n‐Type Bi_2_S_3_ ,” Journal of Electronic Materials 48 (2019): 503–508, 10.1007/s11664-018-6741-4.

[83] X. Yang , J. Guo , Z.‐Y. Wang , et al., “Highly enhanced thermoelectric properties of Bi_2_S_3_ via (Se, Cl)‐co doping in hydrothermal synthesis process,” Journal of Alloys and Compounds 922 (2022): 166252, 10.1016/j.jallcom.2022.166252.

[84] W. Ji , X.‐L. Shi , W. Liu , et al., “Boosting the Thermoelectric Performance of n‐type Bi_2_S_3_ by Hierarchical Structure Manipulation and Carrier Density Optimization,” Nano Energy 87 (2021): 106171, 10.1016/j.nanoen.2021.106171.

[85] J. Yang , G. W. Liu , J. N. Yan , X. Z. Zhang , Z. Q. Shi , and G. J. Qiao , “Enhanced the thermoelectric properties of n‐type Bi_2_S_3_ polycrystalline by iodine doping,” Journal of Alloys and Compounds 728 (2017): 351–356, 10.1016/j.jallcom.2017.08.148.

[86] W. Wang , S. J. Luo , C. Xian , et al., “Enhanced Thermoelectric Properties of Hydrothermal Synthesized BiCl_3_/Bi_2_S_3_ Composites,” J Inorg Mater 34 (2019): 328–334.

[87] J. Guo , Z.‐Y. Wang , L. Chen , et al., “Bi 0.33 (Bi 6 S 9 )Br compositing in Bi 2 S 3 bulk materials forwards high thermoelectric properties,” Physical Chemistry Chemical Physics 24 (2022): 24290–24295, 10.1039/D2CP02805K. 36172840

