# Supplementary material for: Porous Bi2S3 Bulk With Excellent Thermoelectric Performance by Solid States Replacement and Low Melting‐Point Metal Volatilization
Source: Adv Mater. 2026 Jan 2;38(11):e21215. doi: 10.1002/adma.202521215 (PMC12921355; doi:10.1002/adma.202521215)
Supplement: Supplementary file 1 — Supporting File: adma71998‐sup‐0001‐SuppMat.docx [file ADMA-38-e21215-s001.docx]

Supporting Information

Porous Bi_2_S_3_ Bulk with Excellent Thermoelectric Performance by Solid States Replacement and Low Melting-point Metal Volatilization

Zi-Yuan Wang, Jun Guo, Yi-Xin Zhang, Hao Liang, Xing Yang, Rafal E. Dunin-Borkowski, Fengshan Zheng, Lei Jin*, Jing Feng*, and Zhen-Hua Ge*

Z.-Y.Wang, J. Guo, Y.-X. Zhang, H. Liang, X. Yang, J. Feng, Z.-H. Ge

Faculty of Material Science and Engineering and National & Local Joint Engineering Laboratory of Advanced Metal Solidification Forming and Equipment Technology, Kunming University of Science and Technology, Kunming 650093, China

E-mail: jingfeng@kust.edu.cn; zge@kust.edu.cn

Z.-Y. Wang, J. Feng, Z.-H. Ge

Southwest United Graduate School, Kunming 650092, China

R. E. Dunin-Borkowski, L. Jin

Ernst Ruska-Centre for Microscopy and Spectroscopy with Electrons, Forschungszentrum Jülich GmbH, 52425 Jülich, Germany

E-mail: l.jin@fz-juelich.de

F. Zheng

Center for Electron Microscopy, South China University of Technology, Guangzhou 511442, China

**1. Experimental Section**

Materials synthesis: The synthesis process of Bi_2_S_3_ nanorods pretreated with hydrochloric acid has been described in detail in previous reports.^[1]^ According to the nominal composition of FeCoNi (denote FCN), a suitable amount of Fe, Co and Ni powders were weighed and placed in a ball mill tank filled with protective gas. Pure phase FCN was obtained by high energy ball milling at 900 rpm for 2 h. According to the nominal composition of Bi_2_S_3_ + *x* wt.% FCN (*x* = 0, 0.125, 0.25, 0.5, 1.0), the appropriate amount of Bi_2_S_3_ powder and FCN powder were dispersed in ethanol, and the two ethanol suspensions were mixed uniformly by ultrasonic mixing for 5 min. The suspension was then centrifuged and dried, and the obtained powder was loaded into a graphite mold with an inner diameter of 20 mm and sintered at 773 K and 40 MPa for 5 min by spark plasma sintering (SPS) to obtain a disk. The synthesis route of the sample is shown in the schematic diagram (Figure S1).


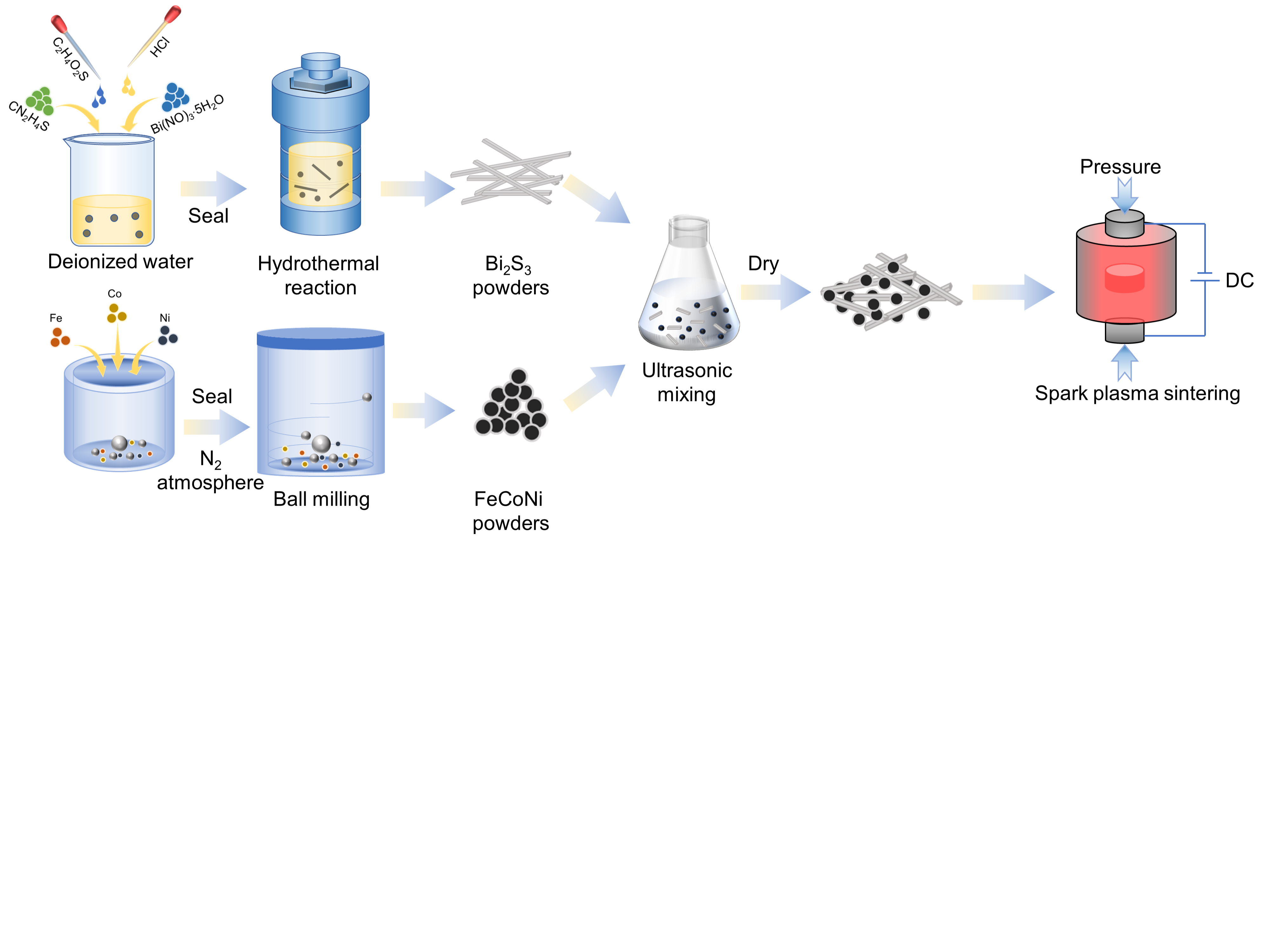


**Figure S1.** Schematic diagram of the synthesis process.

Phase structure and microstructure characterization: X-ray diffraction (XRD) patterns were recorded on a Miniflex600 system operating at 40 kV and 15 mA using monochromatic Cu Kα1 radiation. The morphologies of all samples were characterized by field emission scanning electron microscopy (SEM; Zeiss Merlin, Germany). Scanning transmission electron microscopy (STEM) was utilized to analyze the microstructure of the bulk sample using an FEI Titan 80-200 ChemiSTEM microscope, running at 200 kV, equipped with an ultra-bright field emission source, an aberration corrector for the probe forming lens and a super-X energy dispersive X-ray spectrometer. The convergence and collection semi-angles for high-angle annular dark-field (HAADF) imaging are 25 and 70 – 200 mrad, respectively. Electron probe micro-analysis (EPMA, Shimadzu 1730H) was performed to determine the compositions of the sintered bulk samples.

Thermoelectric performance characterization: The sintered samples were cut and ground into discs and bars with dimensions of 6 mm × 1 mm and 3mm × 3 mm × 12 mm, respectively, for the measurement of the thermal diffusivity coefficient and electrical properties, respectively. The Seebeck coefficient *S* and electrical resistivity were measured from 323 to 773 K using a Seebeck coefficient/electrical resistance measuring system (ZEM-3, Ulvac-Riko, Japan). The thermal conductivity *κ* was calculated using the relationship *κ* = *DρC*_p_, where *D* is the thermal diffusivity coefficient measured by laser thermal conductivity instrument (LFA-467, Netzsch, Germany), *C*p is the specific heat, and *ρ* is the sample density, measured by the Archimedes drainage method. The Hall coefficients (*R*_H_) from 323 K to 773K of samples were measured using a Hall Effect Measurement instrument (Lake Shore 8400, USA). The electrical and thermal properties are measured along the same direction, which is perpendicular to the applied pressure direction during SPS.

Debye-Callaway model calculation: The relaxation time $\tau$ associated with the phonon-phonon U-process (U), phonon-phonon N-process (N), grain/phase boundaries (GB), point defects (PD), dislocations (DS) and precipitates (P) is given by:

$$\tau_{U}^{-1}\approx\frac{\hbar\gamma^{2}}{Mv^{2}\theta_{D}}\omega^{2}Texp(-\frac{\theta_{D}}{3T})$$

$$\tau_{N}^{-1}\approx\beta\tau_{U}^{-1}$$

$$\tau_{GB}^{-1}=\frac{v}{L}$$

$$\tau_{PD}^{-1}=\frac{V_{0}\omega^{4}}{4\pi v^{3}}\sum_{i} x_{i}\left[ \left( \frac{\Delta M}{M} \right)^{2}+\varepsilon\left( \frac{\Delta\alpha}{\alpha} \right)^{2} \right]$$

$$\tau_{D}^{-1}\approx N_{d}\frac{V_{0}^{\frac{4}{3}}}{v^{2}}\omega^{3}+AB_{d}^{2}\gamma^{2}\omega\left\{ \frac{1}{2}+\frac{1}{24}\left( \frac{1-2v}{1-v} \right)^{2}\left[ 1+\sqrt{2}\left( \frac{v_{l}}{v_{t}} \right)^{2} \right]^{2} \right\}$$

$$\tau_{P}^{-1}=v\left( \sigma_{s}^{-1}+\sigma_{l}^{-1} \right)^{-1}V_{p}$$

$$\sigma_{s}=2\pi R^{2}$$

$$\sigma_{l}=\pi R^{2}\frac{4}{9}\left( \frac{\Delta D}{D} \right)^{2}\left( \frac{\omega R}{V} \right)^{4}$$

All relevant parameters and the values used are summarized in Table S3.

**2. Supporting Tables**

**Table S1.** Elemental composition (in mol.%) in the Bi_2_S_3_ matrix in the Bi_2_S_3_ + 0.25 wt.% FCN sample, measured from locations marked by 1 to 4 in Figure S4.

| mol.% | 1# | 2# | 3# | 4# |
| --- | --- | --- | --- | --- |
| Bi | 45.32 | 47.64 | 46.88 | 44.69 |
| S | 54.61 | 52.28 | 53.02 | 55.07 |
| Fe | 0.05 | 0.02 | 0.05 | 0.20 |
| Co | 0.01 | 0.04 | 0.02 | 0.03 |
| Ni | 0.01 | 0.02 | 0.03 | 0.01 |

**Table S2.** Density of Bi_2_S_3_ + *x* wt.% FCN (*x* = 0, 0.125, 0.25, 0.5, 1) samples measured by Archimedes method.

| Sample | Density (g/cm^3^) | Relative density (%) |
| --- | --- | --- |
| *x* = 0 | 5.70 | 83.8 |
| *x* = 0.125 | 5.86 | 86.1 |
| *x* = 0. 25 | 5.95 | 87.3 |
| *x* = 0. 5 | 6.01 | 88.2 |
| *x* = 1 | 6.11 | 89.8 |

Note: The change in relative density reflects the competition between "liquid-phase assisted sintering" (densification) and "Bi volatilization" (pore formation). Here, the increasing trend thus indicates that densification dominates. As discussed in the main text, the metallic Bi generated by the FCN reaction is liquid at sintering temperatures. This liquid phase acts as an effective sintering aid and lubricant, promoting grain rearrangement and mass transfer under pressure. This process significantly eliminates the larger pores formed by the stacking of nanorods, thereby improving grain connectivity. Although the volatilization of nanoscale Bi creates new nanopores, their contribution to the total volume is much smaller than the volume reduction achieved by eliminating the large stacking-induced pores. Consequently, the overall density increases with increased FCN content.

**Table S3.** Parameters for calculating *κ*_lat_ of the Bi_2_S_3_ + 0.25 wt.% FCN sample based on the Debye-Callaway model.

| Parameter | Symbol (unit) | Value |
| --- | --- | --- |
| Grüneisen parameter of matrix | *γ* | 1.66 |
| Average sound velocity of matrix | *v* (m/s) | 1538 |
| Debye temperature of matrix | *θ*_D_ (K) | 156 |
| Average mass of an atom of matrix | *M* (kg) | 1.71×10^-25^ |
| Ratio of normal phonon scattering to Umklapp scattering | *β* | 3.0 |
| Average grain size | *L* (m) | 3.0×10^-7^ |
| Averge atomic volume | *V*_0_ (m^3^) | 2.52×10^-29^ |
| Dislocation density | *N*_d_ (m^-2^) | 8×10^13^ |
| Magnitude of Burgurs vector | *B*_d_ (m) | 7×10^-9^ |
| Longitudinal phonon velocity | *v*_l_ (m/s) | 2504 |
| Transverse phonon velocity | *v*_t_ (m/s) | 1538 |
| Average radius of nanoscale precipitates | *R* (m) | 3×10^-8^ |
| Mass density of matrix | *D* (kg/m^3^) | 6037 |
| Number density of nanoscale precipitates | *V*_p_ (m^-3^) | 5.3×10^14^ |

**3. Supporting Figures**


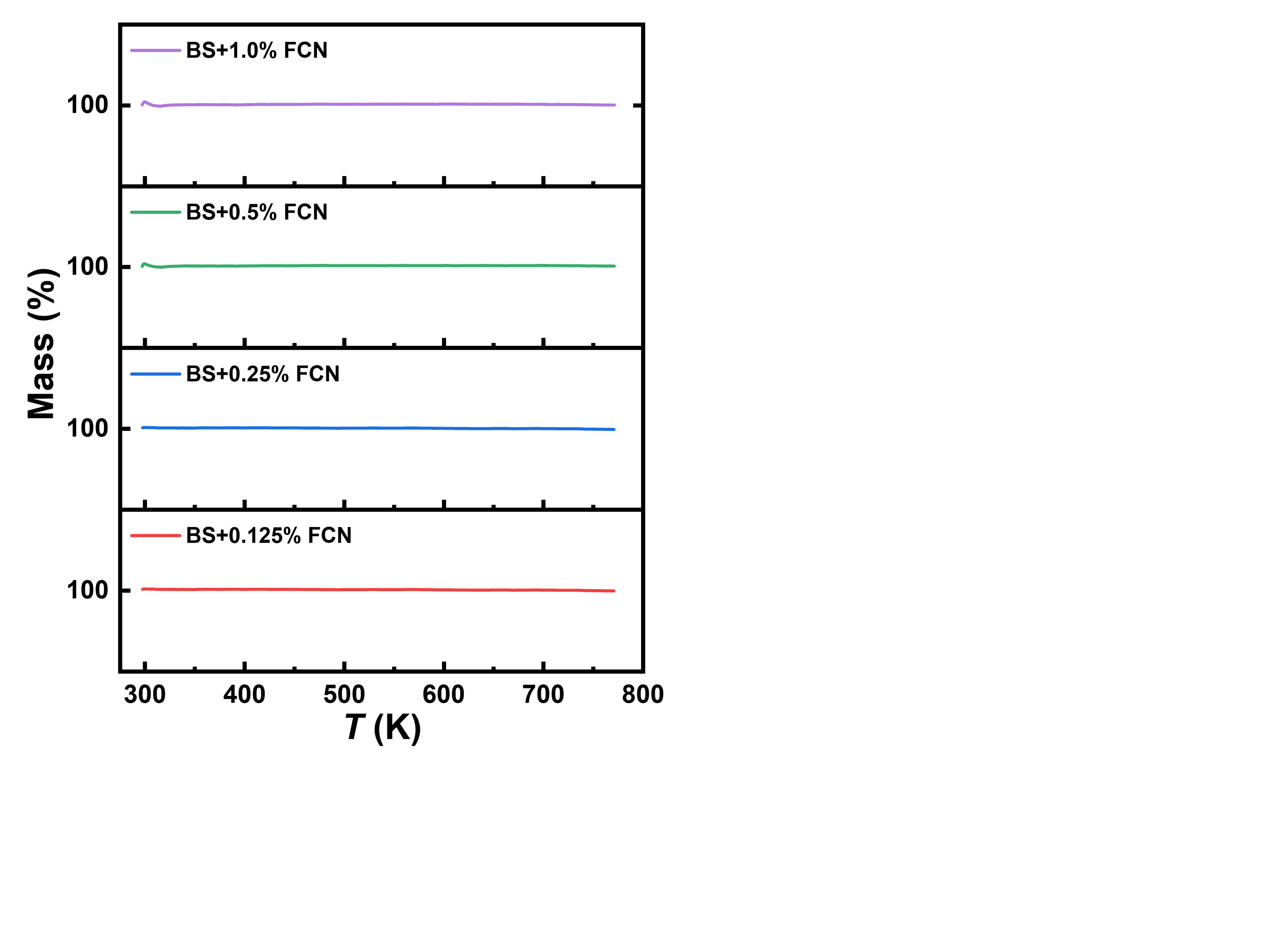


**Figure S2.** Thermogravimetric results of Bi_2_S_3_ + *x* wt.% FCN (*x* = 0.125, 0.25, 0.5, 1.0) bulk samples.


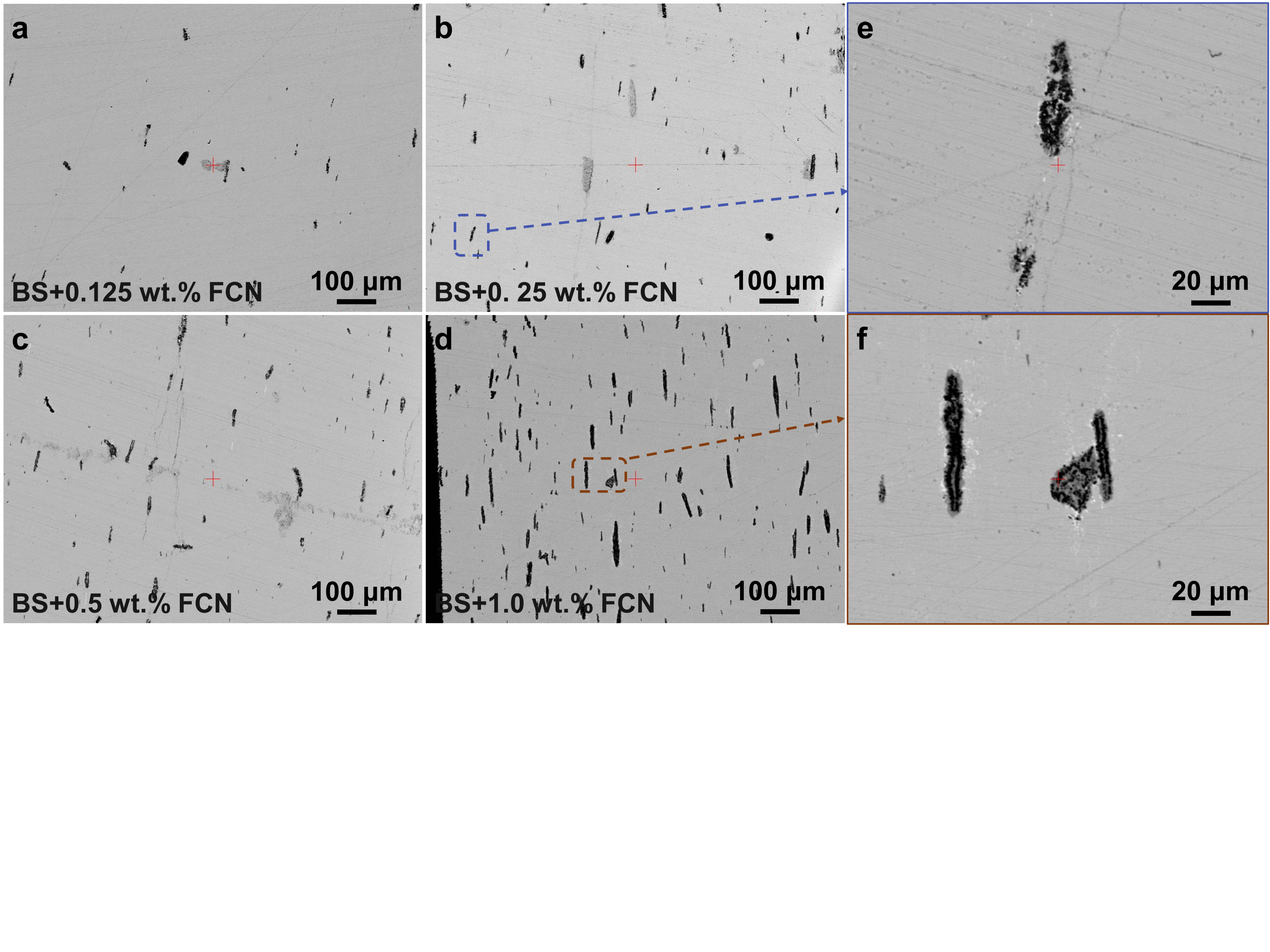


**Figure S3.** Backscatter electron SEM image of Bi_2_S_3_ + *x* wt.% FCN bulk samples. Sample surface is polished for measurement. (a) *x* = 0.125, (b) *x* = 0.25, (c) *x* = 0.5 and (d) *x* = 1.0. (e) and (f) are magnified images of the marked areas in (b) and (d), respectively. BS represents Bi_2_S_3_ for short.


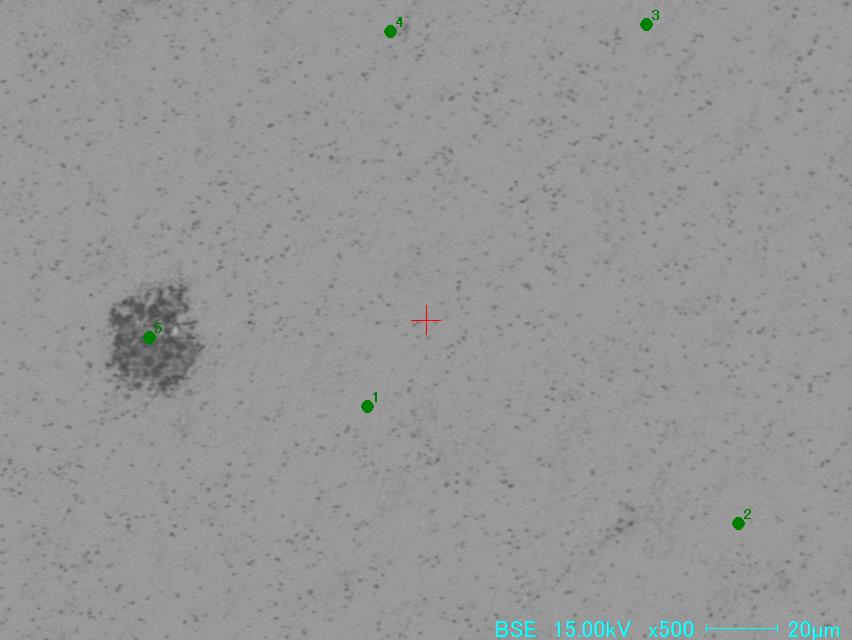


**Figure S4.** EPMA BSE image of the Bi2S3 + 0.25 wt.% FCN sample. The numerical markers 1 to 4 indicate the specific locations within the grey matrix region selected for point analysis. The elemental composition of the selected location is shown in Table S1.


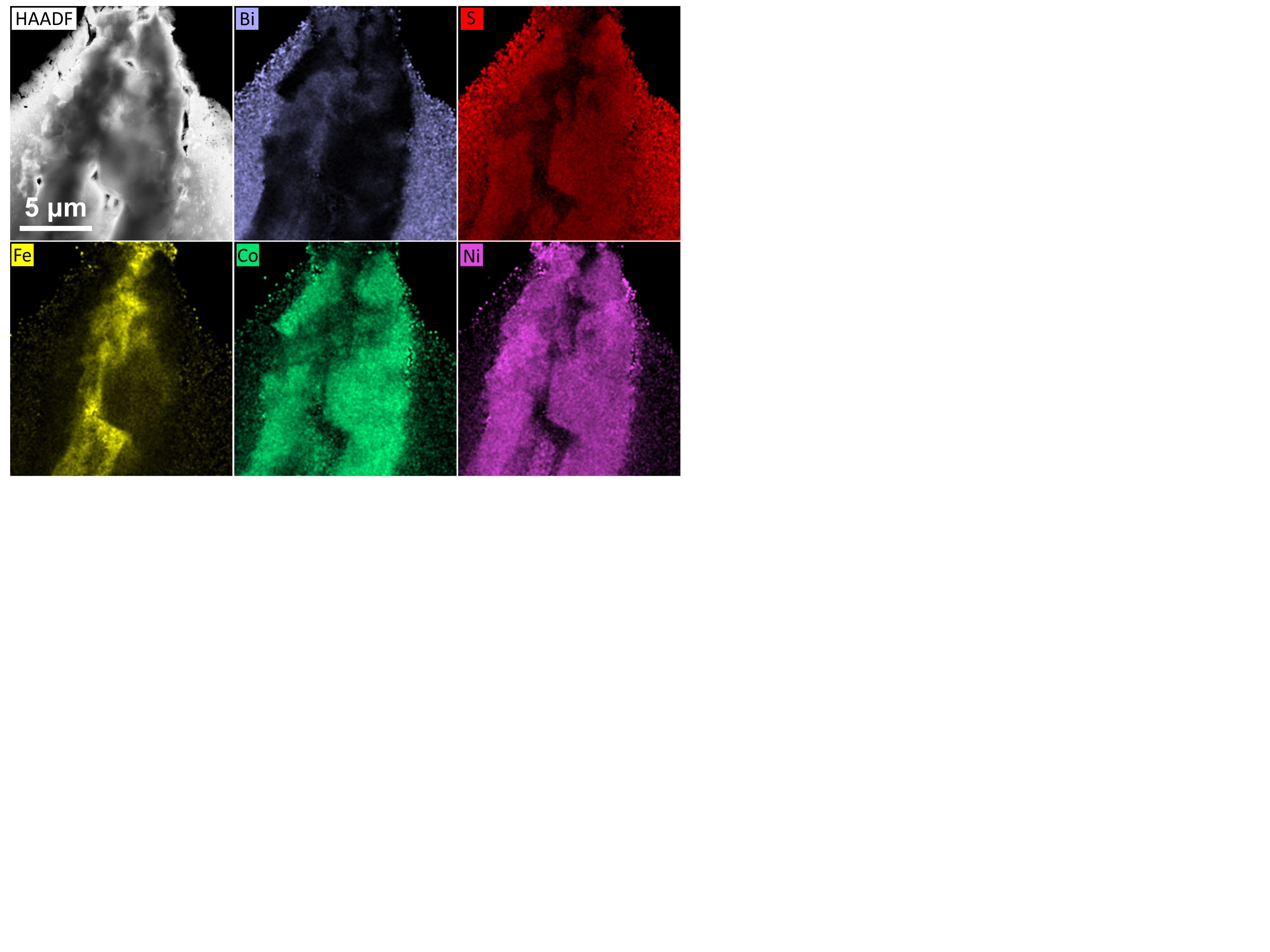


**Figure S5.** HAADF STEM image of the Bi_2_S_3_ + 0.25 wt.% FCN sample and the corresponding energy dispersive X-ray spectroscopy results.


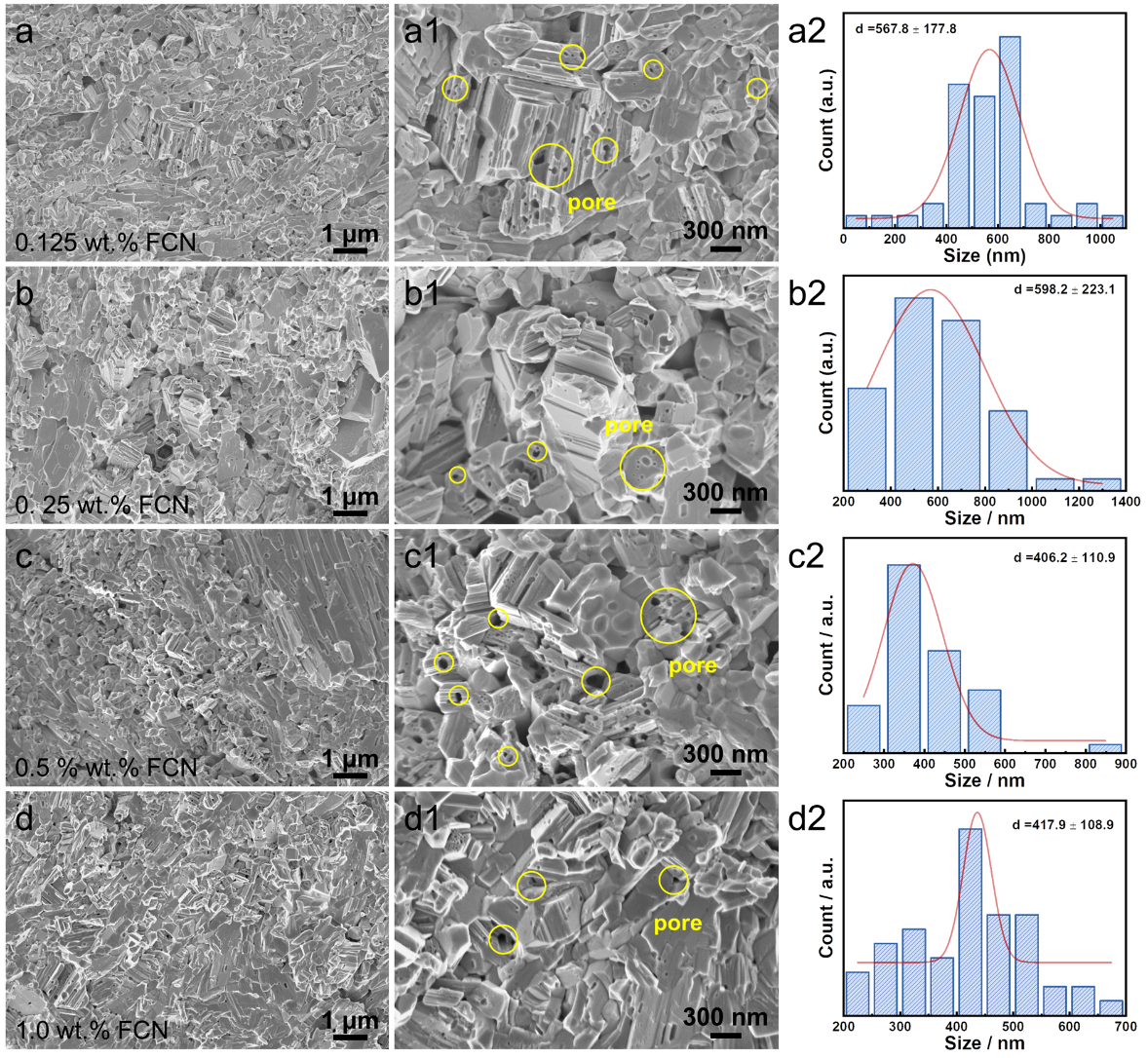


**Figure S6.** SEM images of the fracture surface morphology and grain size of Bi_2_S_3_ + *x* wt.% FCN (*x* = 0.125, 0.25, 0.5, 1.0) bulk samples. (a) 0.125 wt.% FCN; (b) 0.25 wt.% FCN; (c) 0.5 wt.% FCN; (d) 1.0 wt.% FCN sample.


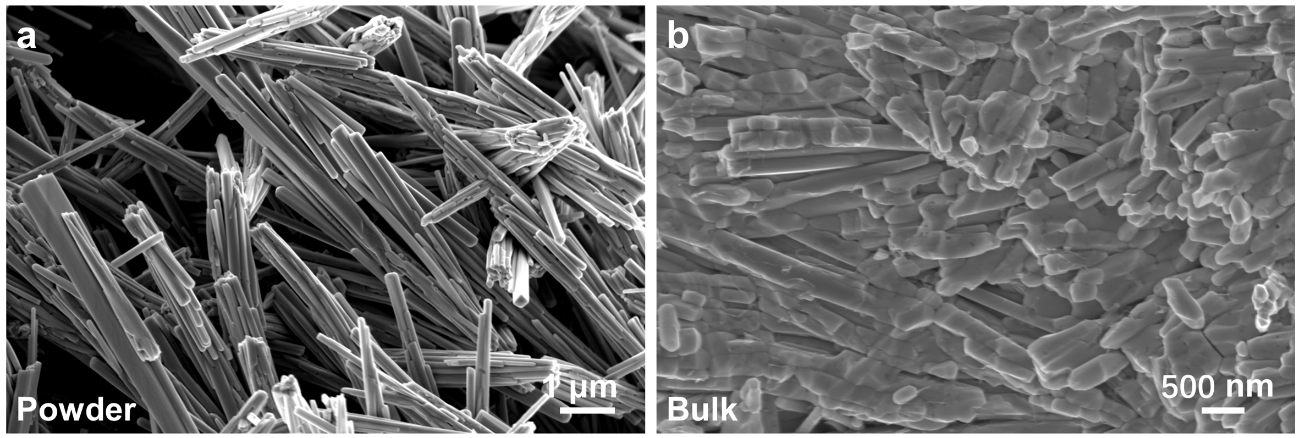


**Figure S7.** SEM images of (a) Bi_2_S_3_ nanorod powder and (b) the fracture surface of bulk Bi_2_S_3_ pure sample (*i.e.*, Bi_2_S_3_ + *x* wt.% FCN, *x* = 0).


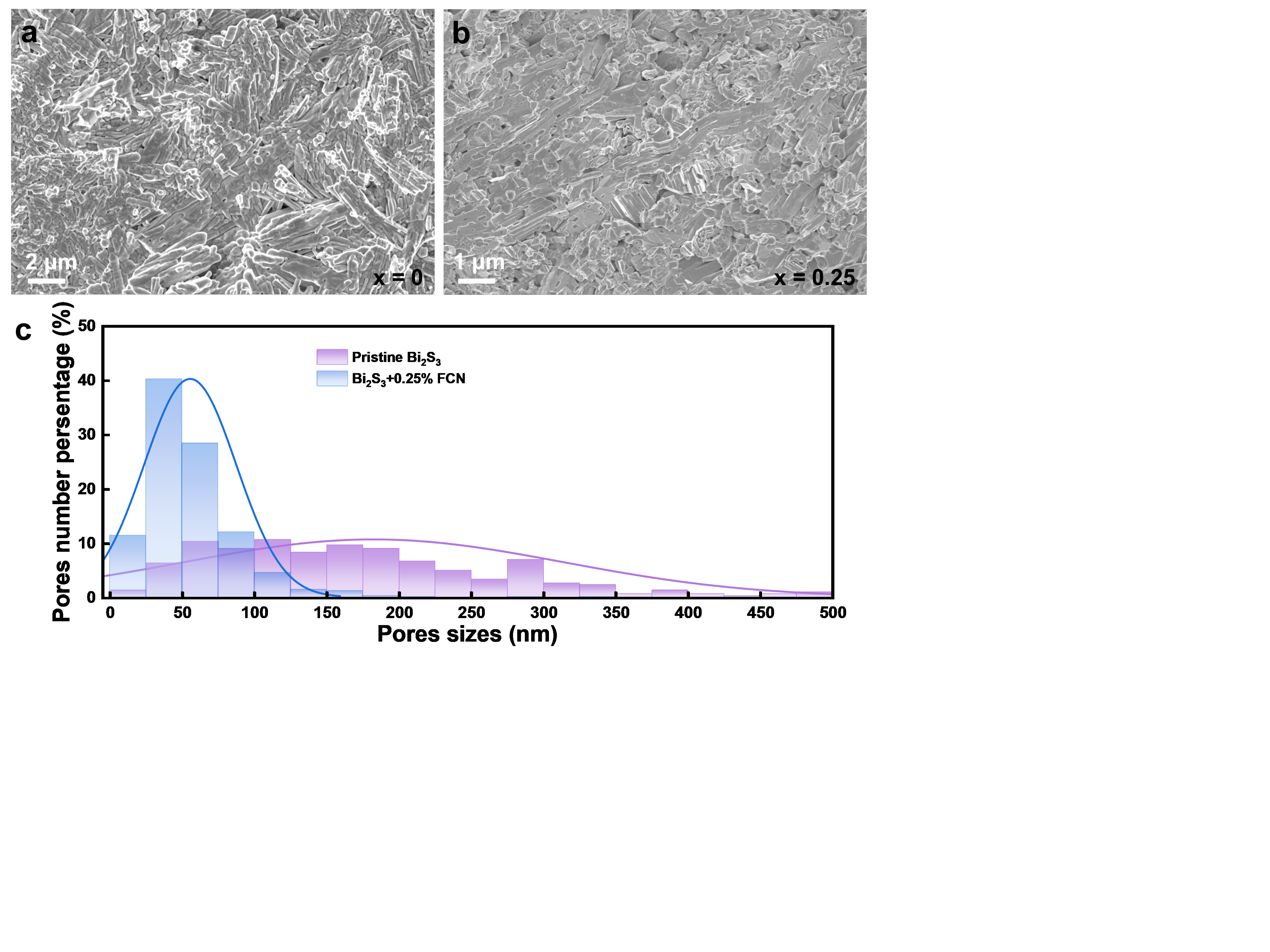


**Figure S8.** SEM images and corresponding pore size statistics of pure samples and Bi_2_S_3_ + 0.25 wt.% FCN samples.


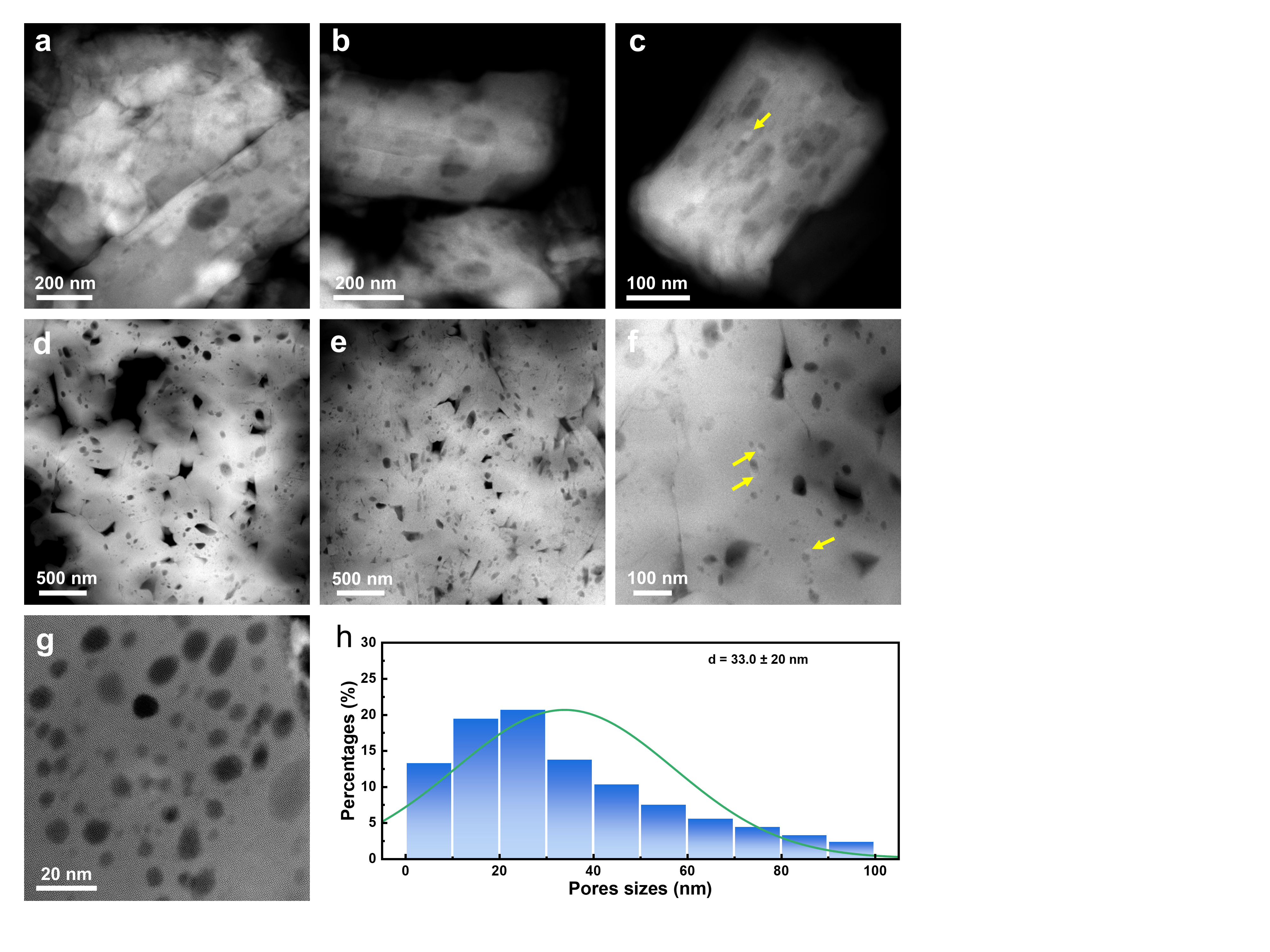


**Figure S9.** (a-c) HAADF STEM images of the Bi_2_S_3_ + 0.25 wt.% FCN sample prepared by crushing a bulk sample into particles; (d-g) HAADF STEM images of the Bi_2_S_3_ + 0.25 wt.% FCN sample prepared by conventional STEM preparation method, including grinding, dimpling and argon ion milling; (h) Statistical results of the pore size distribution in the grain matrix. Yellow arrows mark the residual Bi particles.


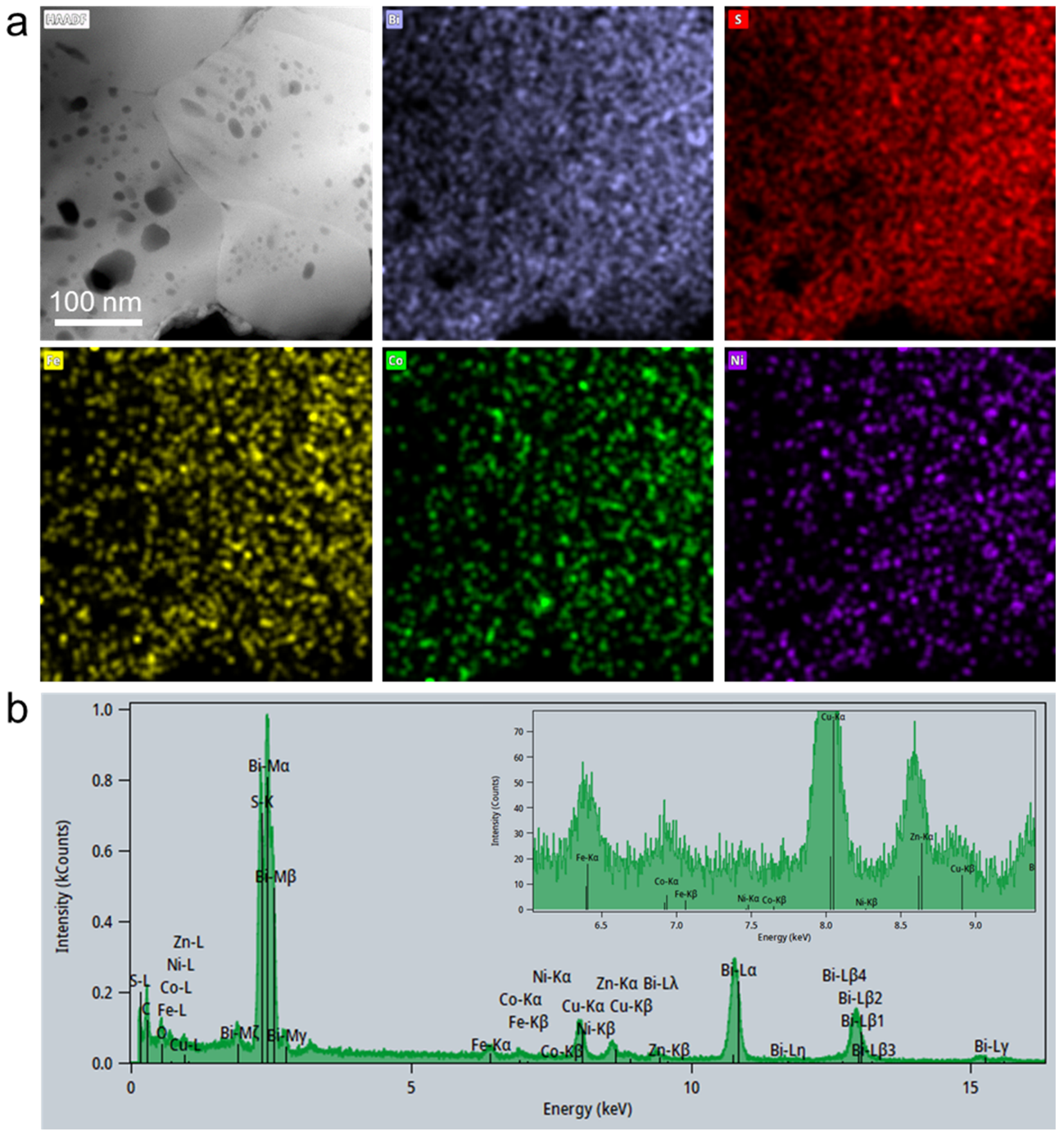


**Figure S10.** (a) HAADF STEM image and corresponding elemental maps of Bi, S, Fe, Co and Ni, respectively. (b) Energy dispersive X-ray spectrum from the entire area of (a). The Cu and Zn signals are from the brass ring used for STEM sample preparation. C is induced by sample contamination during prolonged data acquisition and O may be induced by oxidization during STEM sample preparation.


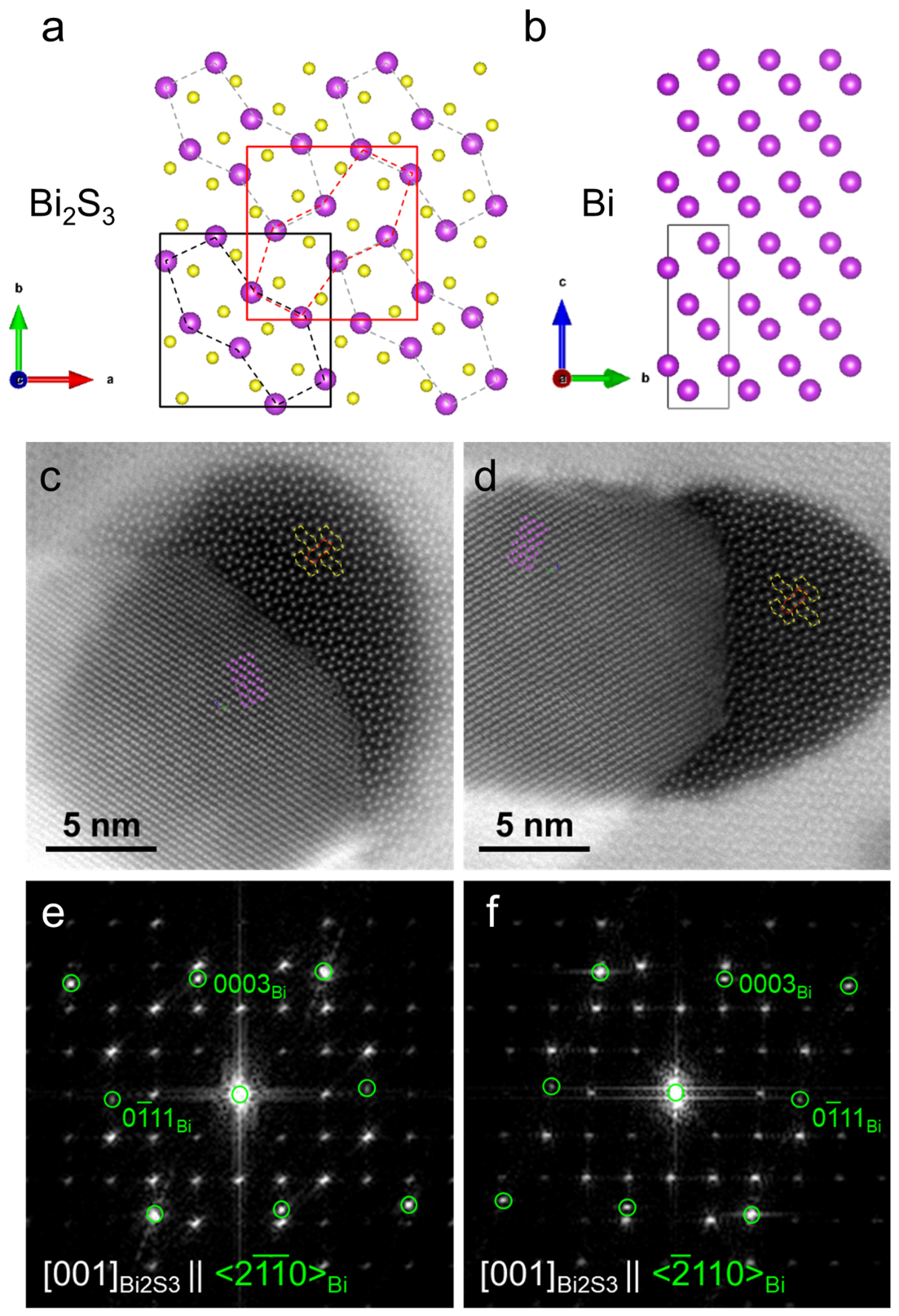


**Figure S11.** Atomic model of (a) Bi_2_S_3_ and (b) Bi. (c, d) Atomic-resolution HAADF STEM images of the Bi_2_S_3_ + 0.25 wt.% FCN bulk sample and (e, f) corresponding fast Fourier transfer patterns, respectively, showing the orientation relationships between Bi_2_S_3_ and Bi.


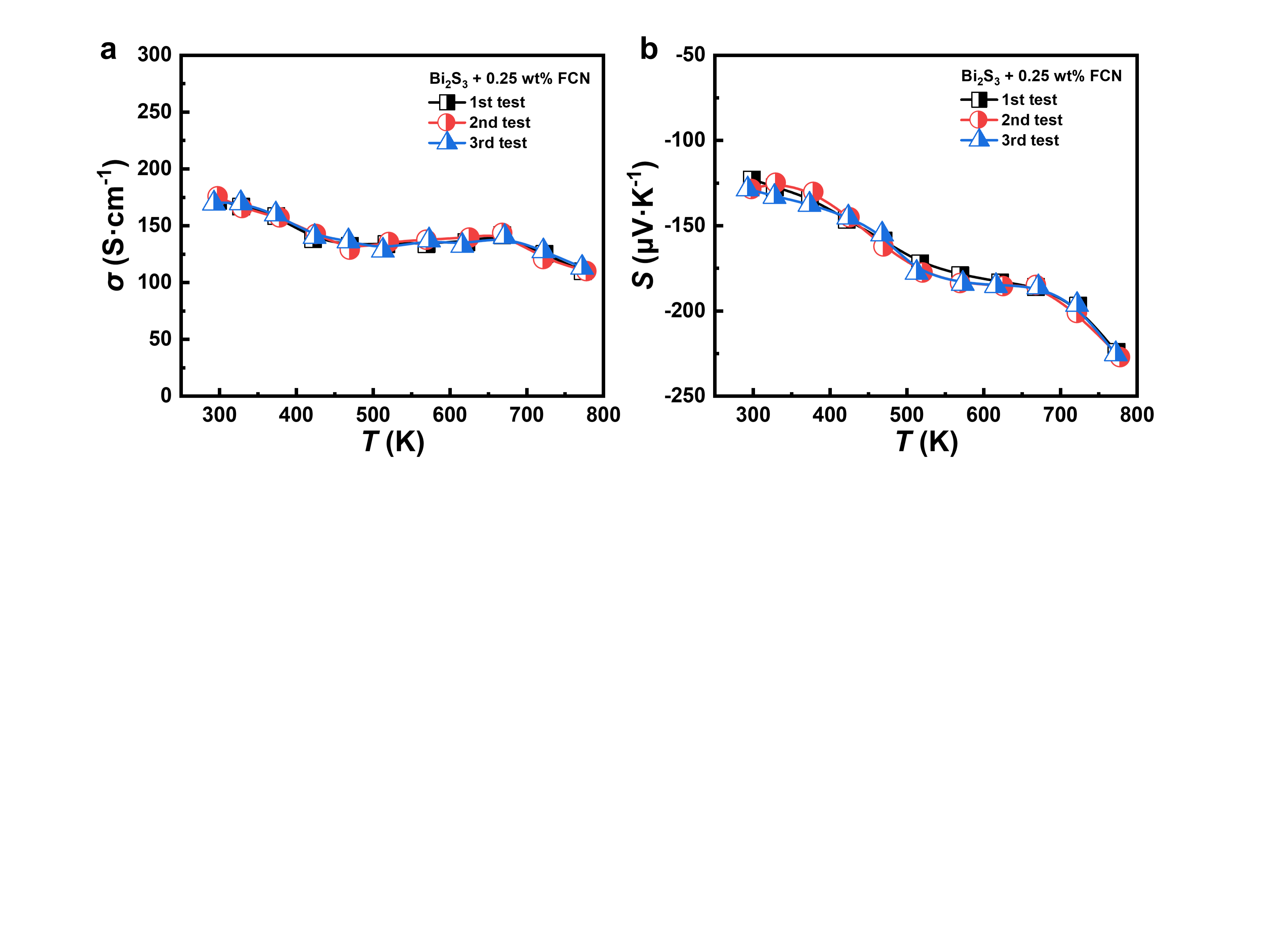


**Figure S12.** Repeatability test results of the Bi_2_S_3_ + 0.25 wt.% FCN sample. (a) Electrical conductivity and (b) Seebeck coefficient.


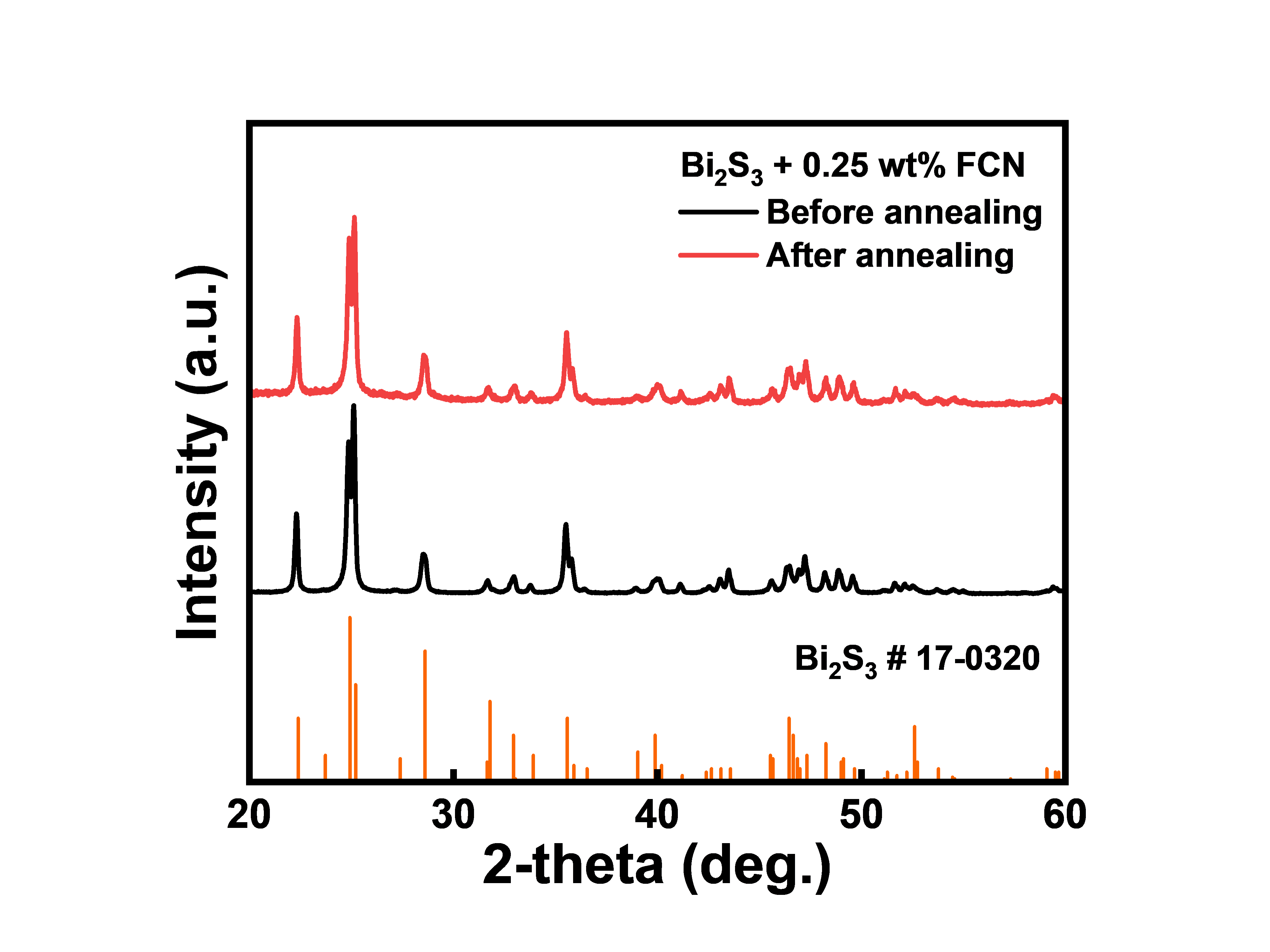


**Figure S13.** XRD patterns of the Bi_2_S_3_ + 0.25 wt.% FCN sample before and after annealing. The annealling was conducted at 773 K in a vacuum for 12 h.


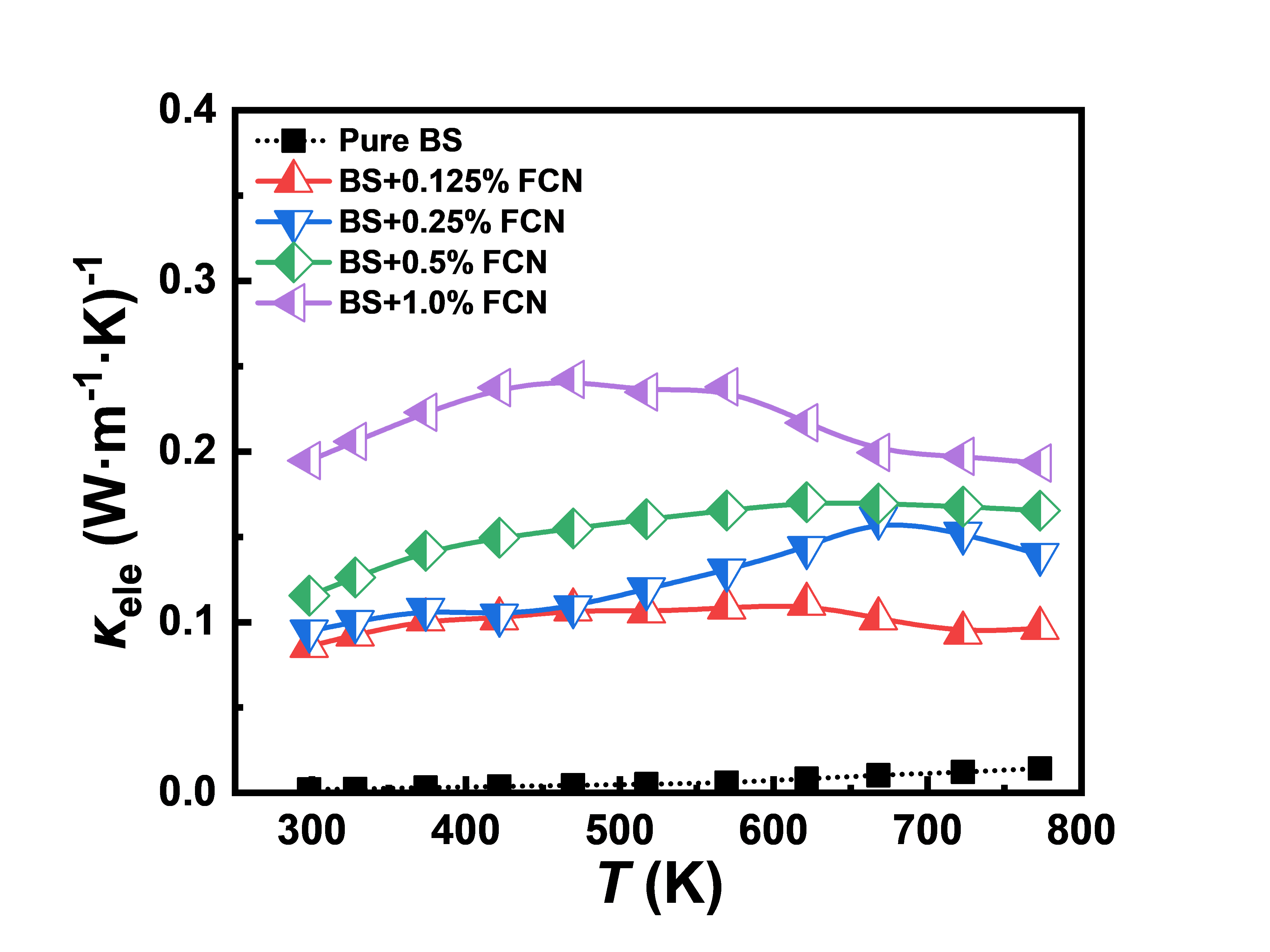


**Figure S14.** Electronic thermal conductivity *κ*_ele_ of Bi_2_S_3_ + *x* wt.% FCN (*x* = 0, 0.125, 0.25, 0.5, 1.0) bulk samples. BS represents Bi_2_S_3_ for short.


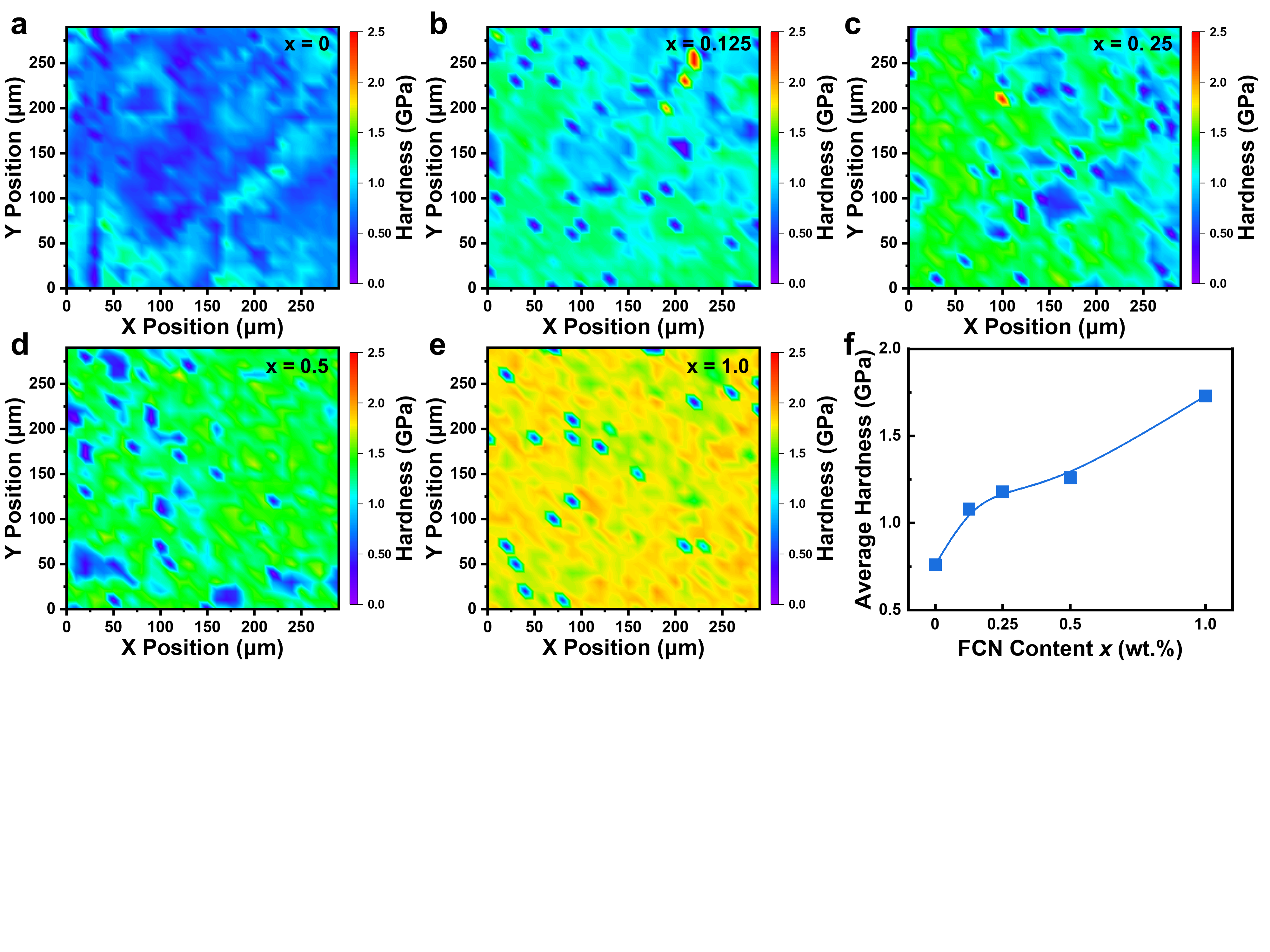


**Figure S15.** Hardness characterization of Bi_2_S_3_ + *x* wt.% FCN (*x* = 0, 0.125, 0.25, 0.5, 1.0) bulk samples.


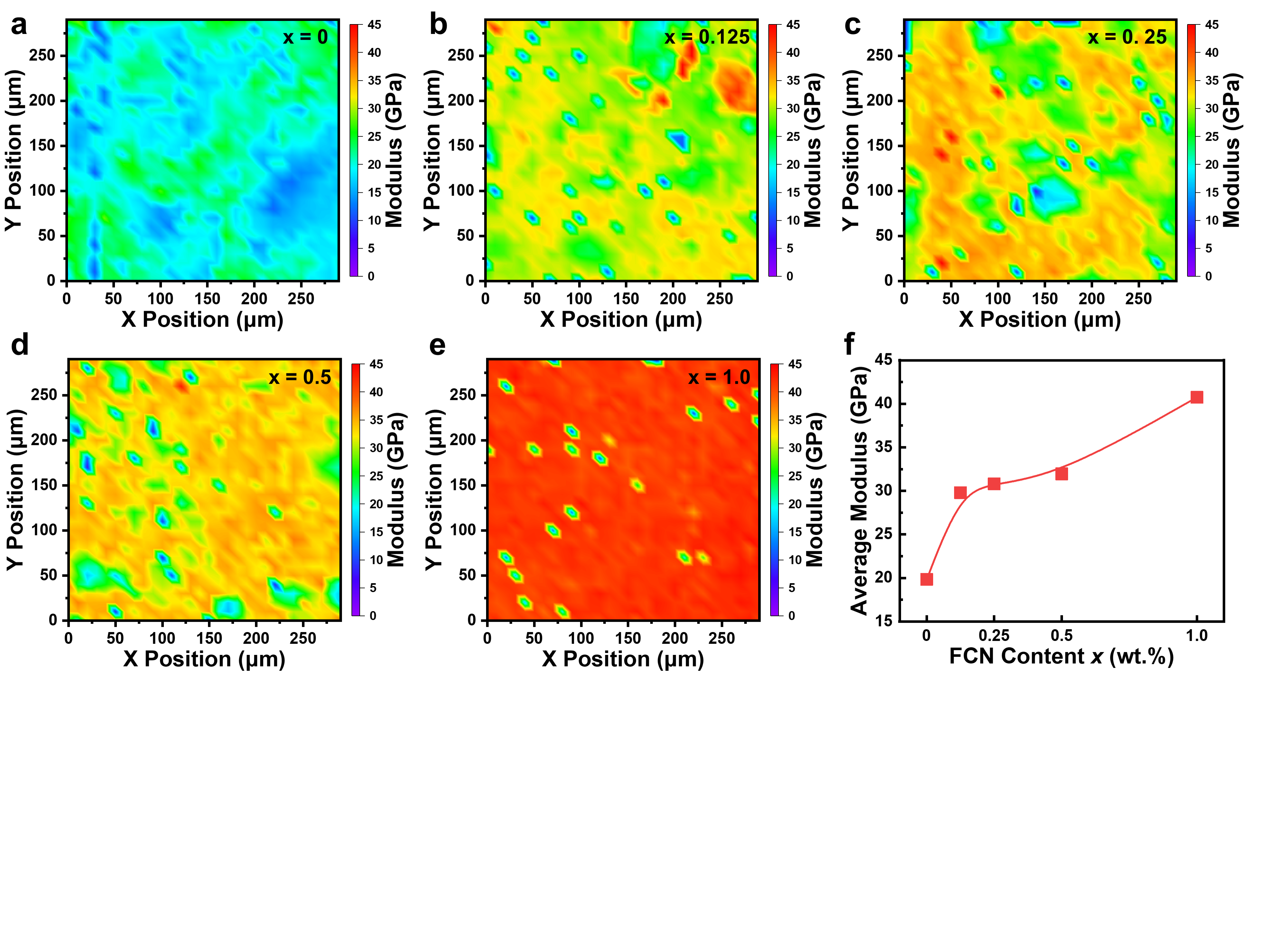


**Figure S16.** Young’s modulus characterization of Bi_2_S_3_ + *x* wt.% FCN (*x* = 0, 0.125, 0.25, 0.5, 1.0) bulk samples.


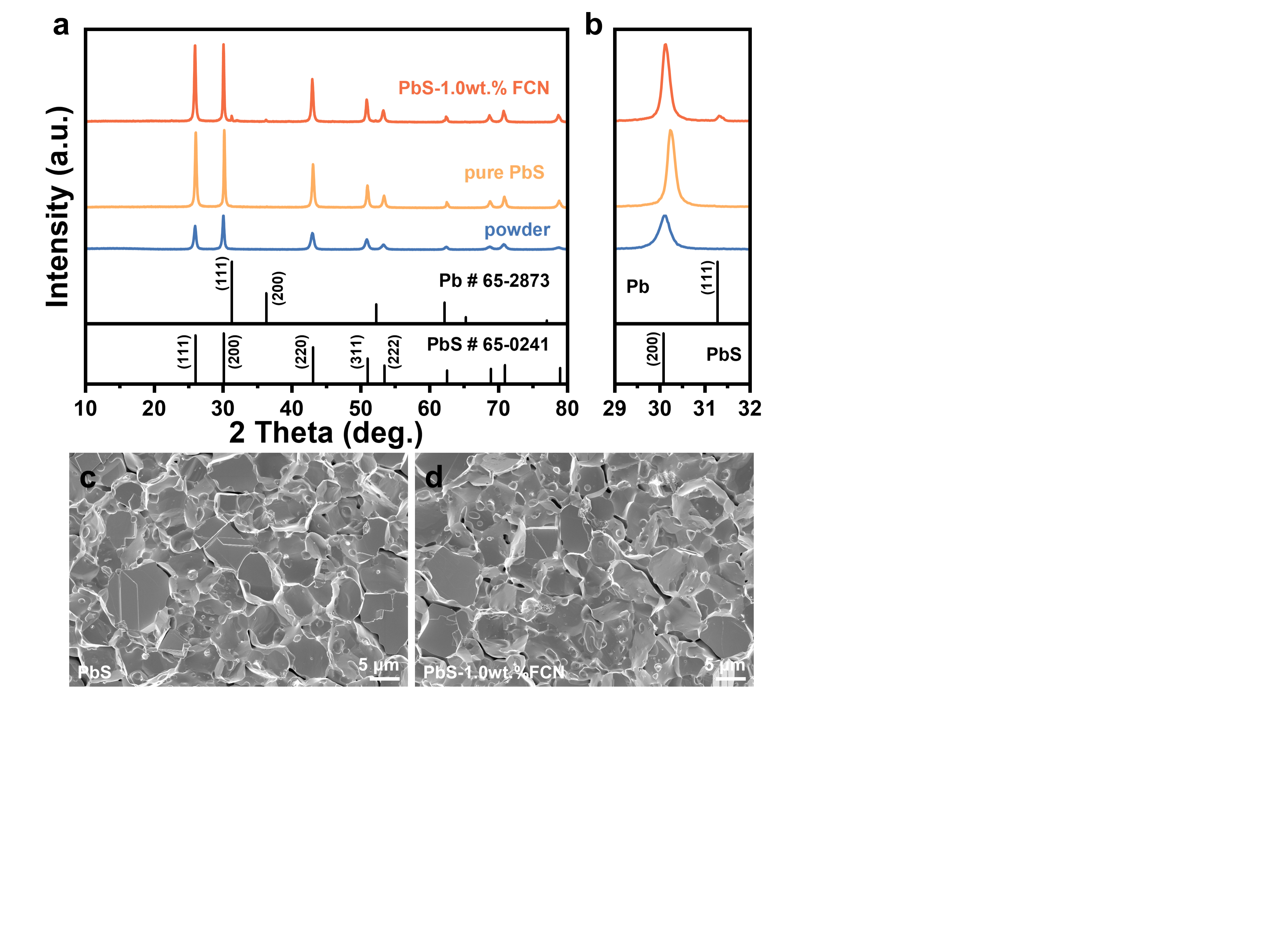


**Figure S17.** (a) XRD and (b) enlarged patterns of PbS + *x* wt.% FCN (*x* = 0, 1) samples. (c) and (d) are fracture surface morphology images of *x* = 0 and *x* = 1 samples, respectively.


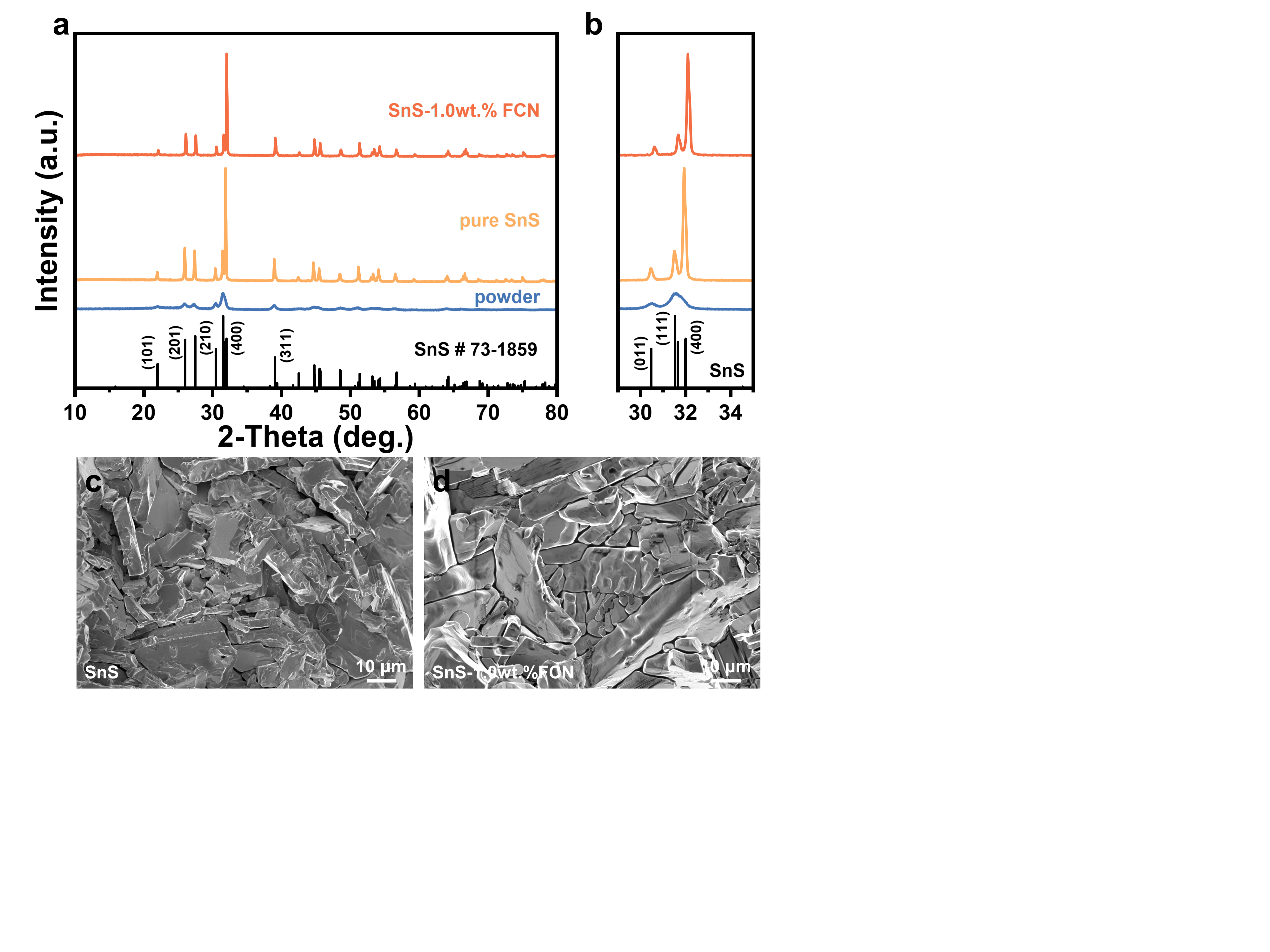


**Figure S18.** (a) XRD and (b) enlarged patterns of SnS + *x* wt.% FCN (*x* = 0, 1) samples. (c) and (d) are fracture surface morphologies of *x* = 0 and *x* = 1 samples, respectively.

**Reference**

[1] J. Guo, J. Yang, Z.-H. Ge, B. Jiang, Y. Qiu, Y.-K. Zhu, X. Wang, J. Rong, X. Yu, J. Feng, J. He, Realizing High Thermoelectric Performance in Earth-Abundant Bi_2_S_3_ Bulk Materials via Halogen Acid Modulation, *Adv. Funct. Mater.* **2021**, *31*, 210838.
